# Caloric restriction promotes resolution of atherosclerosis in obese mice, while weight regain accelerates its progression

**DOI:** 10.1172/JCI172198

**Published:** 2025-07-08

**Authors:** Bianca Scolaro, Franziska Krautter, Emily J. Brown, Aleepta Guha Ray, Rotem Kalev-Altman, Marie Petitjean, Sofie Delbare, Casey Donahoe, Stephanie Pena, Michela L. Garabedian, Cyrus A. Nikain, Maria Laskou, Ozlem Tufanli, Carmen Hannemann, Myriam Aouadi, Ada Weinstock, Edward A. Fisher

**Affiliations:** 1 Department of Medicine, Leon H. Charney Division of Cardiology, Cardiovascular Research Center, New York University Grossman School of Medicine, New York, New York, USA.; 2 Department of Medicine, Section of Genetic Medicine, University of Chicago Pritzker School of Medicine, Chicago, Illinois, USA.; 3 Center for Infectious Medicine, Department of Medicine, Karolinska Institute, Huddinge, Sweden.

**Keywords:** Cardiology, Inflammation, Metabolism, Adipose tissue, Atherosclerosis, Innate immunity

## Abstract

While weight loss is highly recommended for those with obesity, >60% regain their lost weight. This weight cycling is associated with an elevated risk of cardiovascular disease, relative to never having lost weight. How weight loss and regain directly influence atherosclerotic inflammation is unknown. Thus, we studied short-term caloric restriction (stCR) in obese hypercholesterolemic mice, without confounding effects from changes in diet composition. Weight loss promoted atherosclerosis resolution independent of plasma cholesterol. Single-cell RNA sequencing and subsequent mechanistic studies indicated that this can be partly attributed to a unique subset of macrophages accumulating with stCR in epididymal white adipose tissue (eWAT) and atherosclerotic plaques. These macrophages, distinguished by high expression of Fc γ receptor 4 (*Fcgr4*), helped to clear necrotic cores in atherosclerotic plaques. Conversely, weight regain (WR) following stCR accelerated atherosclerosis progression with disappearance of *Fcgr4*^+^ macrophages from eWAT and plaques. Furthermore, WR caused reprogramming of immune progenitors, sustaining hyperinflammatory responsiveness. In summary, we have developed a model to investigate the inflammatory effects of weight cycling on atherosclerosis and the interplay between adipose tissue, bone marrow, and plaques. The findings suggest potential approaches to promote atherosclerosis resolution in obesity and weight cycling through induction of *Fcgr4*^+^ macrophages and inhibition of immune progenitor reprogramming.

## Abstract 2

Weight cycling increases cardiovascular disease. We show benefits in obese mice of short-term caloric restriction on resolving atherosclerosis inflammation, but weight regain accelerates adverse effects.

## Graphical abstract

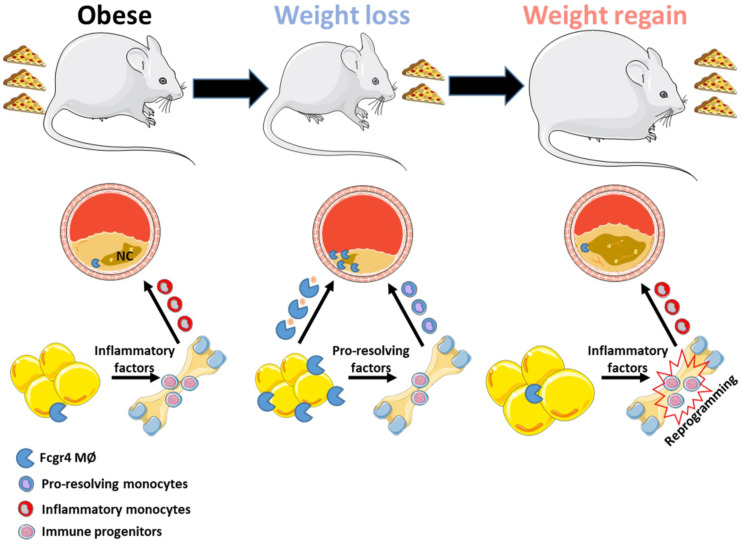

## Introduction

Obesity contributes to the establishment and progression of many diseases, including the leading cause of death, atherosclerotic cardiovascular disease (CVD) (1, 2). A major factor thought to underlie this association is the heightened production and release of a variety of potent inflammatory factors (presumably secreted from visceral adipose tissue), such as IL-6, IL-1β, S100A8/9, and TNF-α, based on observational studies in humans and mouse models (3). Contributing to these inflammatory changes are the effects of obesity on insulin resistance and glucose homeostasis. These metabolic perturbations are potent stimulators of local and systemic inflammation through, for example, the production of reactive oxygen species, ligands for the receptor of advanced glycation end products, and activation of macrophages. In mouse studies, it has also been shown that obesity has effects on hematopoiesis (4). This appears to be through IL-1β secretion from visceral adipose tissue (VAT) macrophages, which is promoted by neighboring adipocytes. This subsequently stimulates the proliferation of bone marrow precursors of monocytes and neutrophils (the major mediators of innate inflammatory responses), thereby increasing their abundance in circulation and VAT (4).

Sustained weight loss can decrease the risk or severity of many obesity-associated diseases, including CVD (5–7). In addition to improving insulin sensitivity and glucose homeostasis (8), weight loss decreases leukocytosis and inflammatory marker expression associated with obesity (9, 10). Furthermore, weight regain (WR; which occurs in >60% of dieters) increases clinical CVD risk (11) and adipose tissue inflammation in mouse models (12) over what is observed in those never having lost weight. However, cellular and molecular immune mechanisms that facilitate resolution of obesity-related inflammation with caloric restriction (CR) and heighten inflammation with WR are not completely known.

To begin addressing these gaps, we previously performed (13) single-cell RNA sequencing (scRNA-Seq) of epididymal white adipose tissue (eWAT; a surrogate in mice for human VAT) because of the above noted association between this depot and adverse consequences of obesity on inflammation (14). We profiled eWAT leukocytes of mice fed a high-fat high-cholesterol (HFHC) diet to induce obesity before and after short-term (2 weeks) reduction of caloric intake by 30% (13). By maintaining HFHC feeding throughout the study and reducing caloric intake, we achieved weight loss while avoiding confounding the data by inducing epigenetic changes in monocytes and their precursors related to diet compositional changes that result by shifting to chow (15, 16). Our results demonstrated that this type of CR (referred to here as short-term CR [stCR]) induced a unique immune milieu in the eWAT, neither totally resembling the obese nor the lean landscape.

Most notable was finding an adipose tissue macrophage (ATM) population that accumulates in eWAT with stCR and is characterized by high expression of the IgG antibody receptor Fc γ receptor 4 (*Fcgr4*) (in humans, *Fcgr3a*), which promotes phagocytosis (17). This ATM population expressed many other genes also implicated in phagocytosis, so we hypothesized that these cells assist in clearing apoptotic cells, products of shrinking adipose tissue, and, indeed, there was evidence of this (13). Clearance of apoptotic cells by macrophages (termed “efferocytosis”) is a critical process in inflammation resolution (18–20), and we hypothesized that stCR induces this process in other macrophage-rich tissues and thereby promotes inflammation resolution beyond that in eWAT (13, 21–26). Thus, in this report, we have focused on effects of stCR on atherosclerotic plaques and extended the results to WR.

## Results

### stCR in obese mice promotes atherosclerosis resolution.

We previously showed in multiple mouse models with hypercholesterolemia that substantial lipid lowering results in the resolution of atherosclerosis, as judged by decreased content and inflammatory properties of macrophages (e.g., 24–26). To isolate the effects of weight loss in obesity on established atherosclerosis, a model in which cholesterol levels are not dramatically affected was required. Another consideration in study design was that switching the feeding of a high-fat diet (HFD) to normal chow results in a severe reduction in food intake (27) (mimicking long-term fasting), as well as in epigenetic changes in macrophages and their precursors related to differences in diet composition (e.g., 15, 16). Thus, we adapted a protocol of mild stCR (28), keeping the diet composition the same, to investigate the role of a clinically relevant level of reduced caloric intake and subsequent weight loss in inflammation resolution in atherosclerosis, independent of cholesterol lowering.

**Figure 1:**
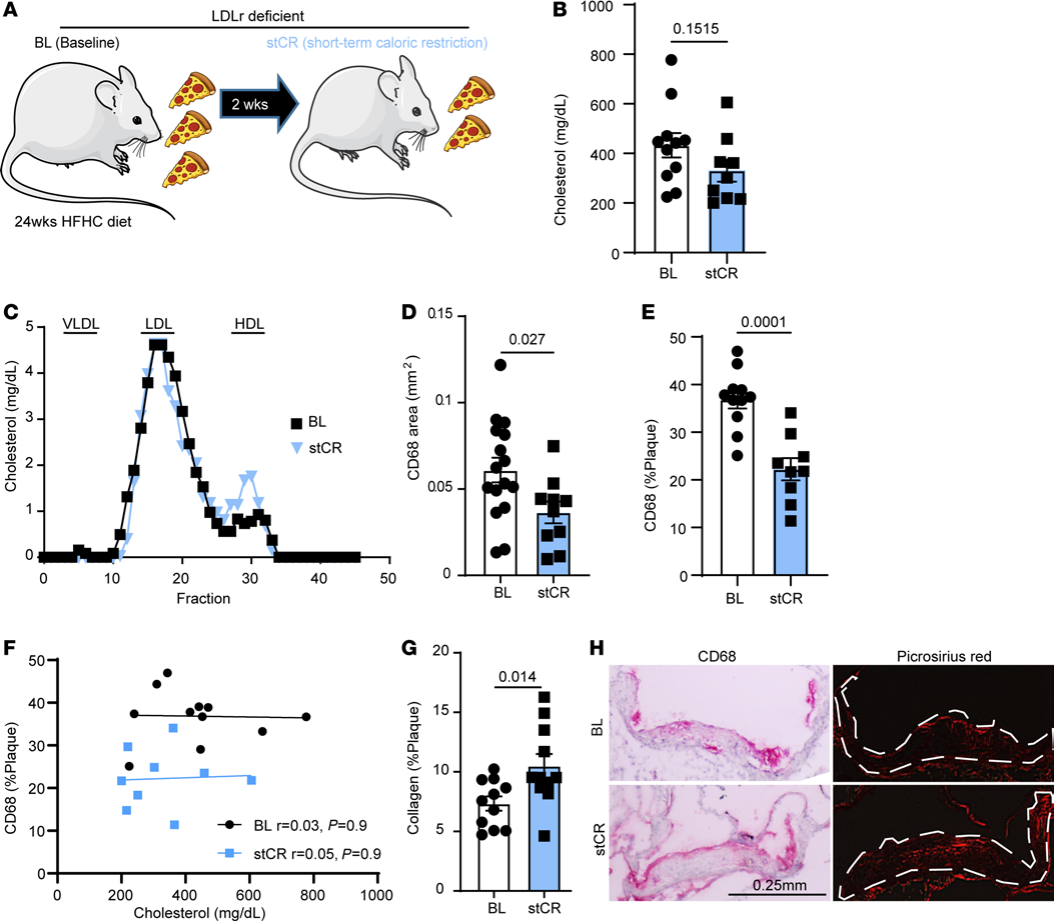
stCR induces atherosclerosis resolution. (**A**) Experimental design. WT mice were fed a HFHC diet and treated with LDLr antisense oligonucleotide for 24 weeks to induce obesity and atherosclerosis (BL group). Mice were then calorically restricted for 2 weeks by reducing food intake by 30% (*n* = 12–14; stCR group). (**B**) Plasma cholesterol levels and (**C**) lipoprotein profile at the end of the experiment. Quantification in (**C**) was performed on pooled plasma from 3 mice/group. (**D** and **E**) Plaque macrophage content quantified through CD68 staining. (**F**) Simple linear regression showing lack of correlation between cholesterol levels and CD68 content in plaques. (**G**) Collagen quantification in plaques assessed through picrosirius red staining. (**H**) Representative aortic root images. Scale bar: 0.25 mm. Data are shown as the mean ± SEM. *P* values were determined via 2-tailed Student’s *t* test.

Thus, WT mice were treated with *Ldlr* antisense oligonucleotide to induce LDL receptor (LDLr) deficiency (as we described previously) (29) and fed a HFHC diet ad libitum for 24 weeks to develop obesity and advanced atherosclerotic plaques. Tissues from the baseline (BL) group (i.e., mice after 24 weeks of HFHC diet) were harvested. To examine early changes induced by weight loss, the stCR group was switched to daily feeding of 70% of their ad libitum consumption of the same HFHC diet for an additional 2 weeks (Figure 1A). The data show that after 24 weeks of treatment, mice were obese, and after 2 weeks of stCR, they lost 14.3% of their weight (Supplemental Figure 1A; supplemental material available online with this article; https://doi.org/10.1172/JCI172198DS1). Upon harvest, several tissues were weighed and a reduction in eWAT mass was observed (Supplemental Figure 1B) with no significant changes to the masses of inguinal white adipose tissue (iWAT), brown adipose tissue (BAT), liver, or kidney (Supplemental Figure 1, C–F).

Examination of metabolic parameters showed marked improvements with stCR, including reduced fasting glucose (Supplemental Figure 1G), lower HOMA-IR (a measure of insulin resistance; Supplemental Figure 1H), and improved glucose tolerance (Supplemental Figure 1, I and J). Moreover, plasma cholesterol levels remained elevated after stCR, with nonsignificant changes between the 2 groups (Figure 1B). To investigate the lipoprotein distribution of plasma cholesterol, a subset of plasma samples was fractioned by fast protein liquid chromatography (FPLC), and cholesterol was measured in each fraction. The results showed no differences in the cholesterol levels in LDL or VLDL fractions between groups. Though HDL cholesterol (HDL-c) was higher in the stCR group by FPLC (Figure 1C), direct measurements of HDL-c in additional plasma samples showed no difference between BL and stCR mice (Supplemental Figure 1K).

For the evaluation of atherosclerosis, aortic roots were sectioned and investigated for plaque size and composition. While plaque area was comparable between the groups (Supplemental Figure 1L), the stCR group had fewer macrophages, observed both as a decrease in the area of CD68^+^ cells (Figure 1D) and their proportion of the total plaque area (Figure 1E). In an independent analysis, consistent findings were observed from digested aortic arches that were analyzed using flow cytometry. These results showed fewer macrophages in aortic arches of stCR mice compared with the BL group (Supplemental Figure 1M). To further establish that the changes in the macrophage content of atherosclerotic plaques were independent of plasma cholesterol levels, we investigated whether these parameters were correlated. Statistical analysis shown in Figure 1F demonstrated no correlation.

The change in plaque cellular composition without a decrease in area is reminiscent of several of our previous studies (e.g., 26, 30, 31), in which inflammation-resolving plaque properties were the predominant feature, with size less significantly affected as the decrease in plaque macrophages was counterbalanced by collagen enrichment, presumably because the content of matrix metalloprotease–producing (inflammatory) macrophages declined. In human plaques, such depletion of macrophages and enrichment in collagen are taken as signs of increased stability (e.g., 32). To quantify changes in the collagen content of plaques, aortic root sections were stained with picrosirius red, and the positive areas were quantified from polarized light images, which represent collagen I, the most common type in atherosclerotic plaques. Indeed, consistent with our previous data and concurrent with decreased plaque macrophages, there was increased collagen content following stCR (Figure 1G). Representative images of aortic roots stained for CD68 and picrosirius red are presented in Figure 1H. As alluded to above, these compositional changes to plaques are increasingly appreciated as more clinically relevant than plaque size in terms of the risk of plaque rupture and myocardial infarction (33).

### Leukocyte subpopulations in plaques and eWAT dramatically change with stCR.

To investigate at the molecular level how stCR influences the immune compartment in atherosclerotic plaques, first, single-cell suspensions were obtained from aortic arches harvested from mice in both experimental groups. CD45^+^ cells (i.e., all leukocytes) that were viable were sorted, and transcripts of individual cells were sequenced using the 10× Genomics platform (following the method described) (34). Because we have also obtained adipose tissue CD45^+^ single-cell transcriptomic data from the same mice (13), the gene expression profiles from both tissues were merged to identify common subpopulations. Quality control and data filtering are displayed in Supplemental Figure 2A.

**Figure 2:**
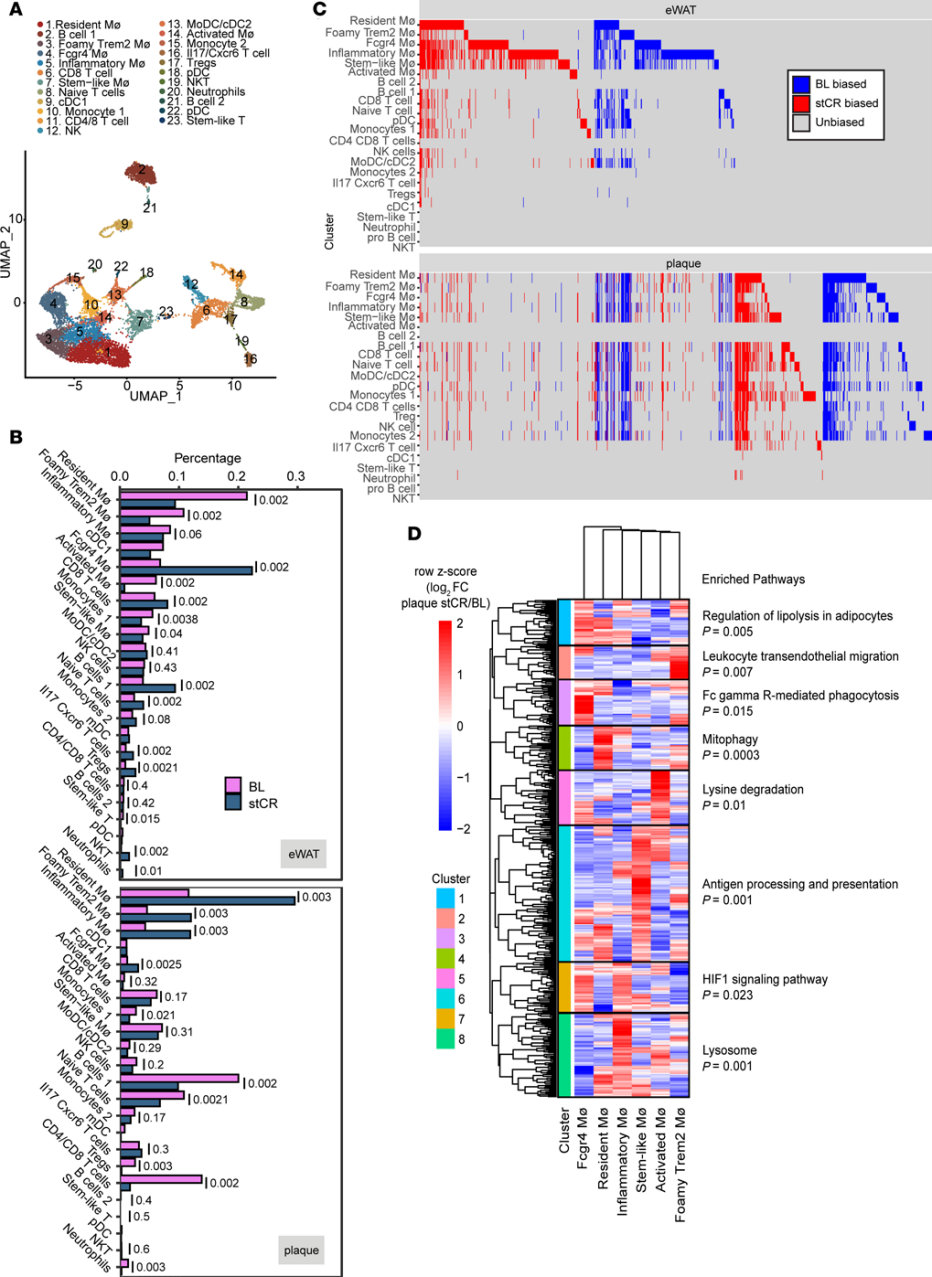
The immune landscape in plaque and eWAT changes with stCR. (**A**) Unbiased clustering of scRNA-Seq dataset represented in an UMAP (uniform manifold approximation and projection). (**B**) Proportion of each cell cluster identified in the scRNA-Seq analysis. (**C**) Genes (columns) differentially expressed in stCR5compared with BL are plotted per cluster (rows) in eWAT (top) and plaques (bottom). Genes that are upregulated in BL and stCR are in blue and red, respectively, or those unchanged are in gray. All DEGs have adjusted *P* value < 0.1. Bias to stCR in eWAT indicates log_2_(stCR/BL) > 0; bias to BL in eWAT indicates log_2_(stCR/BL) < 0; bias to stCR in plaque indicates log_2_(stCR/BL) > 1; bias to BL in plaque indicates log_2_(stCR/BL) < –1. (**D**) Hierarchical clustering of DEGs in all macrophage clusters of the scRNA-Seq dataset and their associated enriched pathways. Values in the heatmap show row *z* scores of log_2_(stCR/BL) in plaque.

Unbiased clustering of the single cells found 23 distinct clusters (Figure 2A and Supplemental Figure 2, B–D). To annotate the different clusters, we used a published meta-analysis of plaque single-cell transcriptomes as a reference dataset (35) (Supplemental Figure 2, C and E). Representative top differentially expressed genes (DEGs) in each cluster are presented in Supplemental Figure 2D and Supplemental Table 1. Many of the clusters in our dataset corresponded to previously published work (35); however, some clusters not found previously were identified as well. For these, we used our previously published dataset from the eWAT CD45^+^ cells (Figure 2A) as the reference dataset (13). Most notable was the appearance of *Fcgr4^+^* macrophages uniquely in our dataset, which was identified due to their preferential accumulation in stCR conditions. Cell proportions were plotted for each tissue in BL and stCR conditions (Figure 2B). Note that while all clusters are shared across eWAT and plaques, their distributions considerably differed in both the obese and stCR conditions.

We also investigated whether obesity and stCR drove similar gene expression in both eWAT and plaques, as well as in distinct leukocyte clusters within each tissue. The expression of each DEG in eWAT was plotted per leukocyte cluster, and its corresponding expression in plaques is shown in Figure 2C. We classified all DEGs (columns) as either BL biased, with log_2_ fold change ≥ 1 higher expression in plaque BL (blue), or stCR biased, with higher log_2_ fold change ≥ 1 expression in stCR (red) across all clusters (rows). Many columns (i.e., DEGs) show a signal in multiple rows (cell clusters), indicating that several clusters differentially express the same genes within each tissue, and often in the same direction (either BL or stCR biased). To look further into this, we plotted the number of clusters that shared DEGs in each tissue (agnostic of whether they are BL or stCR biased). Supplemental Figure 2F shows that most changes in gene expression are restricted to a single cell cluster. Numerous genes, however, were differentially expressed in multiple clusters, with some changing coordinately in >15 (Supplemental Figure 2F and Supplemental Tables 2 and 3).

We next investigated whether genes change concordantly (i.e., undergo expression changes in the same direction in response to stCR treatment) across tissues (Figure 2C). For example, macrophages may have changes resulting from their well-known plasticity in different tissue environments, but there are also likely to be similarities related to the common origin of these cells from circulating monocytes or their response to treatment. When looking at individual genes (columns) across tissues (top and bottom panels), several appear to be similarly changing in both eWAT and plaques (as reflected by showing signals in the same columns in the top and bottom plots). This suggests that a core set of genes (Supplemental Tables 2 and 3) is regulated similarly not only between clusters, but also across tissues (e.g., same column in Figure 2C). Most genes, however, appear to be uniquely differentially expressed in 1 tissue or the other (i.e., not showing concordant signal in Figure 2C), consistent with previous studies (36). Supplemental Table 2 summarizes all DEGs shared by 5 or more clusters, further indicating if the expression is BL or stCR biased and in which tissue.

We also aimed to infer cellular communications between the 23 cell clusters. Ligand–receptor interaction analysis was performed (see Methods), and the number of interacting pairs in plaques and eWAT (Supplemental Figure 2G) was plotted. The color represents the number of ligand–receptor pairs found between clusters in the *x* and *y* axes. Notably, in both plaques and eWAT, the cells with the highest number of significant ligand–receptor interactions among the leukocytes in both BL and stCR groups are the macrophages (such as Foamy-*Trem2* macrophages and activated macrophages), which mostly communicate with other macrophages (Supplemental Figure 2G). In addition, in eWAT of both BL and stCR groups, there were also predicted interactions of inflammatory macrophages with T cells (CD8^+^, Treg, and NKT).

To further explore the responses of each macrophage cluster to stCR, the log fold change (LFC) values of DEGs compared with BL were hierarchically clustered and examined for pathway enrichment (Figure 2D). First, we performed differential expression analysis (see Methods) between stCR and BL within each macrophage cluster and calculated LFC values for those genes in all other macrophage clusters. We then performed hierarchical clustering on the LFC values across genes (rows) and macrophage clusters (columns).

There were 8 well-defined gene clusters capturing distinct patterns of differential gene expression across the macrophage clusters. Gene Ontology and Kyoto Encyclopedia of Genes and Genomes (KEGG) analyses to assess pathway enrichment in each hierarchical cluster were then performed, and the resulting terms are shown in Figure 2D. Interestingly, we have previously observed that *Fcgr4*^+^ macrophages accumulate in the eWAT in obese mice following stCR, and they express many genes associated with phagocytosis (13), a process that would be expected to have important functional effects in both tissues. Analysis of plaque macrophage transcriptome changes with stCR indicated that pathways upregulated in *Fcgr4*^+^ macrophages include “Fc-gamma receptor-mediated phagocytosis” and “regulation of lipolysis in adipocytes” (Figure 2D). Since *Fcgr4*^+^ macrophages were enriched in both plaques and eWAT with stCR, we chose to investigate these cells further and examine whether they play a role in stCR-induced atherosclerosis resolution.

### Fcgr4^+^ macrophages accumulate with weight loss and promote beneficial changes in atherosclerotic plaques.

**Figure 3:**
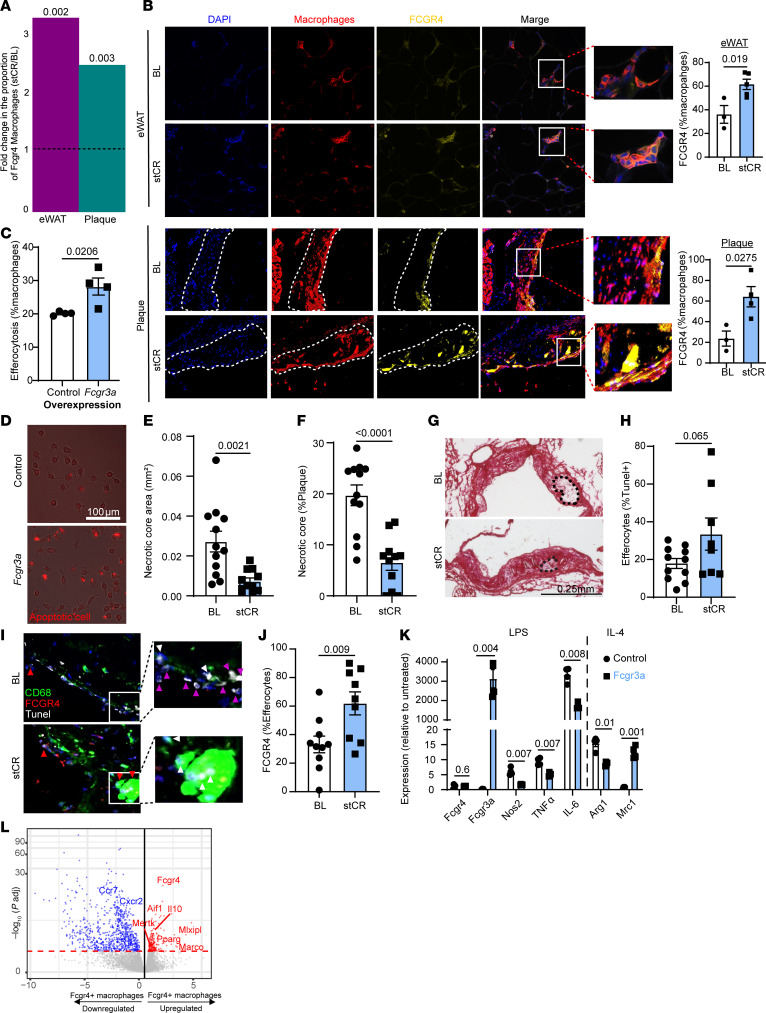
FCGR4^+^ macrophages accumulate with weight loss and promote a pro-reparative phenotype and increased efferocytosis. (**A**) Fold change in the proportion of *Fcgr4*^+^ macrophages in stCR mice, compared with BL, in plaque and eWAT, quantified from the scRNA-Seq data. Dotted line indicates the BL levels, and *P* values (on top of bars) for the differences from BL were determined using false discovery rate. (**B**) Images and quantification of FCGR4 and macrophage staining in eWAT and aortic roots (*n* = 3–5). (**C** and **D**) Human *Fcgr3a* mRNA, or a scrambled sequence as control, was introduced to BMDMs. After 24 hours, macrophages were exposed to fluorescently labeled apoptotic macrophages. Efferocytotic events were determined as macrophages having an attached or engulfed red label. Scale bar: 100 μm. (**E**–**G**) Plaque necrotic core quantification in root sections of BL and stCR mice presented in Figure 1 (*n* = 11–12). Scale bar: 0.25 mm. (**H**–**J**) In situ efferocytosis assay of aortic root sections in which (**I**) apoptotic cells were labeled by TUNEL (white), macrophages by anti-CD68 (green), and FCGR4 (red) and nuclei by DAPI (blue). White arrowheads indicate macrophage-associated TUNEL, purple arrowheads mark free TUNEL, and red arrowheads point to Fcgr4^+^ macrophages associated with TUNEL. Efferocytosis was calculated as (**H**) total efferocytes (TUNEL^+^ macrophages) and as (**J**) FCGR4^+^ and TUNEL^+^ macrophages. (**K**) Gene expression of inflammatory (*Nos2*, *Il6*, and *Tnfa*) and anti-inflammatory (*Mrc1* and *Arg1*) markers following LPS or IL-4 stimulation, respectively, of control or *Fcgr3a*-overexpressing macrophages (*n* = 4). (**L**) Volcano plot showing DEGs in FCGR4^+^ compared with FCGR^–^ macrophages from eWAT following stCR. *P* values were determined by 2-tailed Student’s *t* test. Data are shown as the mean ± SEM.

As just noted, in both plaques and eWAT, stCR increased *Fcgr4^+^* macrophages (Figure 2B and Figure 3A). These transcriptomic results were verified at the protein level using immunofluorescence staining of aortic roots and eWAT sections for macrophages (CD68 and F4/80, respectively) and FCGR4 (Figure 3B).

We hypothesized that the functional consequence of the enrichment would be enhanced tissue repair (specifically, inflammation resolution and favorable tissue remodeling), as suggested by Fc-receptors being potent mediators of phagocytosis (17). Moreover, these macrophages were found to be enriched in other phagocytosis-related genes (13). There is strong rationale for this hypothesis from the recognition in the atherosclerosis field that efferocytosis, or the phagocytosis of dying cells, is an inflammation-resolving process that limits the size of plaque necrotic cores (18). Thus, the fact that *Fcgr4* is increased in stCR conditions made us interested to determine whether this had a functional consequence on efferocytosis.

To mimic this increase in vitro, we measured the efferocytotic activity of macrophages overexpressing the mRNA of the human homolog of *Fcgr4*, *Fcgr3a*. Control cells were similarly treated with a scrambled mRNA sequence. The cells were used in a standard assay (37) in which macrophages are incubated with fluorescent apoptotic cells, with efferocytotic activity quantified by counting the frequencies of macrophages that consume apoptotic cells. *Fcgr3a*-overexpressing macrophages had enhanced efferocytotic capacity compared with control cells (Figure 3, C and D), suggesting that the basal level of expression was limiting. To examine whether stCR induces efferocytosis in vivo, plaque necrotic core size was assessed in aortic root images, which, as alluded to above, has been shown to inversely correlate with macrophage efferocytotic activity (37, 38). Indeed, the data showed smaller necrotic cores in the stCR group (Figure 3, E–G).

To further examine macrophage efferocytotic capacity in vivo, plaques were also assessed as described previously (37) for macrophage-associated apoptotic cells (observed as TUNEL^+^). The results showed that compared with BL mice, plaques in the stCR group tended to have more macrophages associated with apoptotic cells (Figure 3H), again indicative of increased efferocytosis. Notably, we previously reported in eWAT that after stCR, the content of macrophages that had multiple nuclei increased, consistent with their efferocytosis of apoptotic cells (13). Here, we found by immunostaining that in both BL and stCR mice, a sizable proportion of the efferocytes in plaques were FCGR4^+^, with substantially more efferocytes expressing FCGR4 in the stCR group (33% in BL and 62% in stCR plaques; Figure 3, I and J). It should be remembered that each macrophage performing efferocytosis typically clears several apoptotic cells (37–39). Therefore, even a modest increase in macrophages that have enhanced efferocytotic ability can result in a large increase in dead cell removal, consistent with what we have observed with changes in the necrotic core.

We also investigated whether stCR influences the expression of inflammatory and pro-resolving genes by performing flow cytometric analysis of plaque macrophages (Supplemental Figure 3A). This analysis revealed that stCR increased the levels of proteins associated with pro-resolution (e.g., CD163 and CD206) and decreased levels of those associated with inflammation (e.g., Ly6C and CD14). We further tested the response of macrophages overexpressing the human *Fcgr3a* to inflammatory and anti-inflammatory stimuli. To confirm that the *Fcgr3a* overexpression was successful and specific, we measured the expression of the human (*Fcgr3a*) and mouse (*Fcgr4*) genes and found no difference in the expression of *Fcgr4* but a marked increase in *Fcgr3a* (Figure 3K). Upon exposure to the inflammatory stimulus LPS, *Fcgr3a*-overexpressing macrophages showed decreased expression of pro-inflammatory genes, including *Il6*, *Tnfa*, and *Nos2* (Figure 3K). The response to IL-4 was more diverse, as *Fcgr3a*-overexpressing macrophages enhanced the expression of the pro-resolving gene mannose receptor (*Mrc1*), but not Arginase 1 (*Arg1*), as shown in Figure 3K.

We sought to further characterize *Fcgr4^+^* macrophages by performing bulk RNA-Seq on eWAT macrophages that were either FCGR4 positive or negative. Obese mice were subjected to stCR, after which single cells from eWAT were isolated. FCGR4 positive and negative macrophages were flow-sorted and their transcriptomes compared. DEGs between macrophages expressing FCGR4 and those not expressing it are presented in Supplemental Table 4 and Figure 3L. We found that many genes upregulated in FCGR4^+^ macrophages are known to be important in the efferocytosis process, such as *Il10* (40) and *Pparg* (41), adding further evidence of their phagocytic/efferocytotic function. We also queried the significantly up- and downregulated genes (adjusted *P* value < 0.1) of FCGR4^+^ macrophages for KEGG pathway enrichment (Supplemental Figure 3B). Both “response to interferon γ” and “antigen processing and presentation” were enriched in the genes from FCGR4^+^ macrophages, while “extracellular matrix organization,” “regulation of angiogenesis,” and “collagen fibril organization” were enriched in the genes with lower expression in FCGR4^+^ macrophages. Interestingly, genes involved in “cell killing” were enriched in genes both up- and downregulated in FCGR4^+^ macrophages, suggesting that different components of these pathways are at play in FCGR4^+^ macrophages compared with FCGR4^–^ macrophages.

Taken together, these data suggest that stCR induces a desirable environment in both plaques and eWAT that is associated with decreased expression of inflammatory genes, increased expression of pro-resolving genes, and enrichment of *Fcgr4*^+^ macrophages, with these cells promoting clearance of apoptotic cells.

### eWAT-derived Fcgr4^+^ macrophages contribute to the reduction in plaque necrotic core.

The data above suggest that cells in the *Fcgr4*^+^ macrophage cluster promote a reduction in plaque necrotic core upon stCR-induced weight loss. Despite being relatively enriched to similar levels in both plaques and eWAT following stCR (Figure 3A), *Fcgr4*^+^ macrophages, in terms of cell frequency among all leukocytes, constituted a much larger population in eWAT (Figure 2B) (13). Thus, we hypothesized that *Fcgr4*^+^ macrophages in eWAT may contribute to resolving atherosclerotic inflammation by interorgan mechanisms.

**Figure 4:**
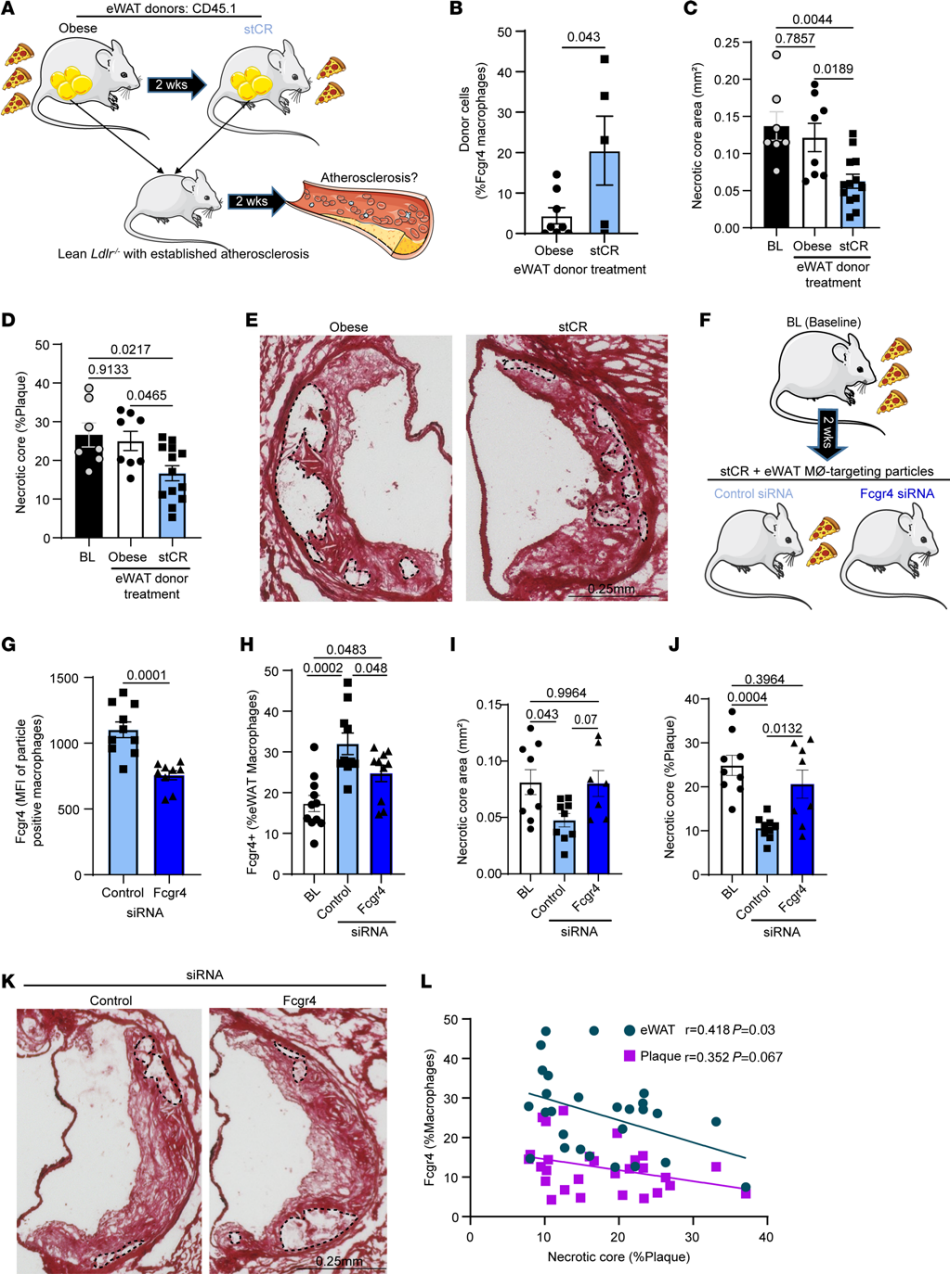
eWAT-derived *Fcgr4*^+^ macrophages reduce plaque necrotic core. (**A**) Schematic of adipose tissue transplantation experiment (*n* = 7–12). (**B**) Presence of FCGR4^+^ macrophages derived from donor adipose tissue in plaques of recipient mice, determined by flow cytometry (*n* = 5–6). (**C** and **D**) Plaque necrotic core quantification in root sections of eWAT recipients, according to donor’s group treatment, with (**E**) representative images. Scale bar: 0.25 mm. (**F**) Experimental design of knockdown of *Fcgr4* in eWAT macrophages during stCR (*n* = 7–10). Flow cytometry analysis after injection of control or *Fcgr4* siRNA particles of (**G**) FCGR4 surface expression in eWAT macrophages and (**H**) FCGR4^+^ macrophages as percent of total eWAT macrophages. (**I** and **J**) Plaque necrotic core quantification in root sections, with (**K**) representative images. Scale bar: 0.25 mm. (**L**) Simple linear regression showing correlation between *Fcgr4^+^* macrophages and necrotic core. *P* values were determined via (**B** and **H**) 2-tailed Student’s *t* test, (**C**, **D**, **G**, **I**, and **J**) 1-way ANOVA with Tukey’s multiple-comparison test, and (**L**) simple linear regression analysis. Data are shown as the mean ± SEM.

To test this hypothesis, we performed adipose tissue transplantation studies, adapting a protocol we previously used (4). Experimentally, 400 mg of eWAT from obese mice pre- or post-stCR was transferred to lean *Ldlr*^–/–^ mice with established atherosclerosis (accomplished as in ref. 42) by low-fat high-cholesterol diet feeding for 20 weeks to avoid confounding effects of obesity. Because we were focused on assessing atherosclerosis resolution, mice were transferred to normal chow (with no added cholesterol) to halt disease progression starting 2 days pre-surgery. To investigate trafficking between adipose tissue and atherosclerotic plaques, we incorporated into the protocol eWAT donors with the congenic pan-leukocyte marker CD45.1 and, as recipients, CD45.2^+^ *Ldlr*^–/–^ mice with atherosclerosis (Figure 4A). Two weeks post-eWAT transplantation, aorta were harvested and examined by flow cytometry and immunohistochemistry. Single-cell suspensions from aortic arches were analyzed by flow cytometry for leukocyte populations that originated from the transferred eWAT (i.e., CD45.1^+^).

Approximately 3% of plaque leukocytes were derived from the eWAT in recipients of either obese or stCR eWAT (Supplemental Figure 4A). Despite similar trafficking of ATMs to plaques, there was preferential enrichment of eWAT-derived FCGR4^+^ macrophages in plaques of recipients of stCR eWAT (Figure 4B). This might be due in part to changes in macrophage subpopulation abundances in the donor adipose tissue (Figure 3B), which show increased *Fcgr4*^+^ macrophages in stCR eWAT.

Recipients of both obese and stCR eWAT had comparable plasma cholesterol levels, which were significantly lower, as expected from the diet change, than BL mice (Supplemental Figure 4B). Histological analyses of aortic root sections showed similar plaque size and macrophage content in recipients of either obese or stCR eWAT (Supplemental Figure 4, C–E). Importantly, plaque necrotic cores were smaller in the recipients of the stCR adipose tissue compared with both BL mice (no adipose transplantation) and recipients of obese adipose tissue (Figure 4, C–E). This suggested that the source of eWAT regulates plaque necrotic core content, with a contribution related to the trafficking of FCGR4^+^ macrophages. This also implies that FCGR4 was not just a marker of cells that accumulate in eWAT with stCR, but a functional factor in the necrotic core improvement.

To directly test this, we knocked down *Fcgr4* expression in eWAT macrophages during the stCR phase. First, WT mice were injected with *Pcsk9*-AAV.8 to induce LDLr deficiency (43) and fed a HFHC diet to promote the development of both atherosclerosis and obesity. After 24 weeks, mice were randomized to have similar average weight/group, and stCR began for 2 weeks. During the stCR phase, mice received daily i.p. injections of glucan shell particles containing siRNA to *Fcgr4* or a scrambled sequence as control (Figure 4F). Note that these particles are preferentially taken up by ATMs when obese mice are injected i.p. (44).

Pilot studies showed that the particles were taken up by 38% of eWAT and 4% of iWAT macrophages but were not detected in the spleen or bone marrow (Supplemental Figure 4F). *Fcgr4* siRNA-containing particles decreased FCGR4 surface levels in eWAT (Figure 4G) but not plaque macrophages (Supplemental Figure 4G). Flow cytometry analysis showed that compared with BL mice, both groups that underwent stCR had more FCGR4^+^ macrophages in eWAT (Figure 4H), recapitulating our earlier findings (Figure 3). Of the eWAT macrophages that were positive for the particles, FCGR4 levels were 32% higher in the control particle–treated group compared with the *Fcgr4* siRNA particle–treated mice (Figure 4G). This corresponded to a 25% decrease in the abundance of eWAT FCGR4^+^ macrophages in the *Fcgr4* siRNA particle–treated mice (Figure 4H). Despite this downregulation of FCGR4 compared with the control siRNA–treated mice, the *Fcgr4* siRNA particle–treated mice still had significantly higher FCGR4^+^ macrophages compared with BL (Figure 4H), likely due to the strong induction by stCR. This suggests that the results of this experiment represent partial success in suppressing *Fcgr4* mRNA expression and likely underestimate the role of eWAT FCGR4^+^ macrophages in inflammation resolution.

When examining macrophage and necrotic core content in aortic root sections, we found that plaque size (Supplemental Figure 4H) and macrophage content (Supplemental Figure 4, I and J) were similar between the weight loss groups. In contrast, necrotic cores trended to be larger and their proportion of plaque area greater in both BL and recipients of the *Fcgr4* siRNA-containing particles (Figure 4, I–K) compared with control siRNA recipients, despite similar plasma cholesterol levels (Supplemental Figure 4K). Furthermore, there was a negative correlation between the abundance of FCGR4^+^ macrophages in the plaques or eWAT and plaque necrotic core content (Figure 4L). These data indicate that elevated FCGR4 levels in eWAT macrophages help to reduce plaque necrotic core, likely related to their greater efferocytotic capacity (Figure 3 and associated text above). The reduction in necrotic core might be due to trafficking of FCGR4^+^ macrophages to plaques (Figure 4B), or by other mechanisms, such as secreted factors or extracellular vesicles originating from these cells (45, 46).

### WR accelerates atherosclerosis progression and diminishes the content of Fcgr4^+^ macrophages in plaques and eWAT.

Weight loss that is sustained is beneficial in reducing CVD risk (5–7), consistent with the effects of stCR in promoting atherosclerosis inflammation resolution (Figures 1–4). Weight fluctuation, however, is associated with worsening of CVD compared with the maintenance of an obese state (e.g., 11, 47). This is an important clinical issue because maintaining weight loss is extremely challenging, with >60% of patients regaining weight (48, 49).

**Figure 5:**
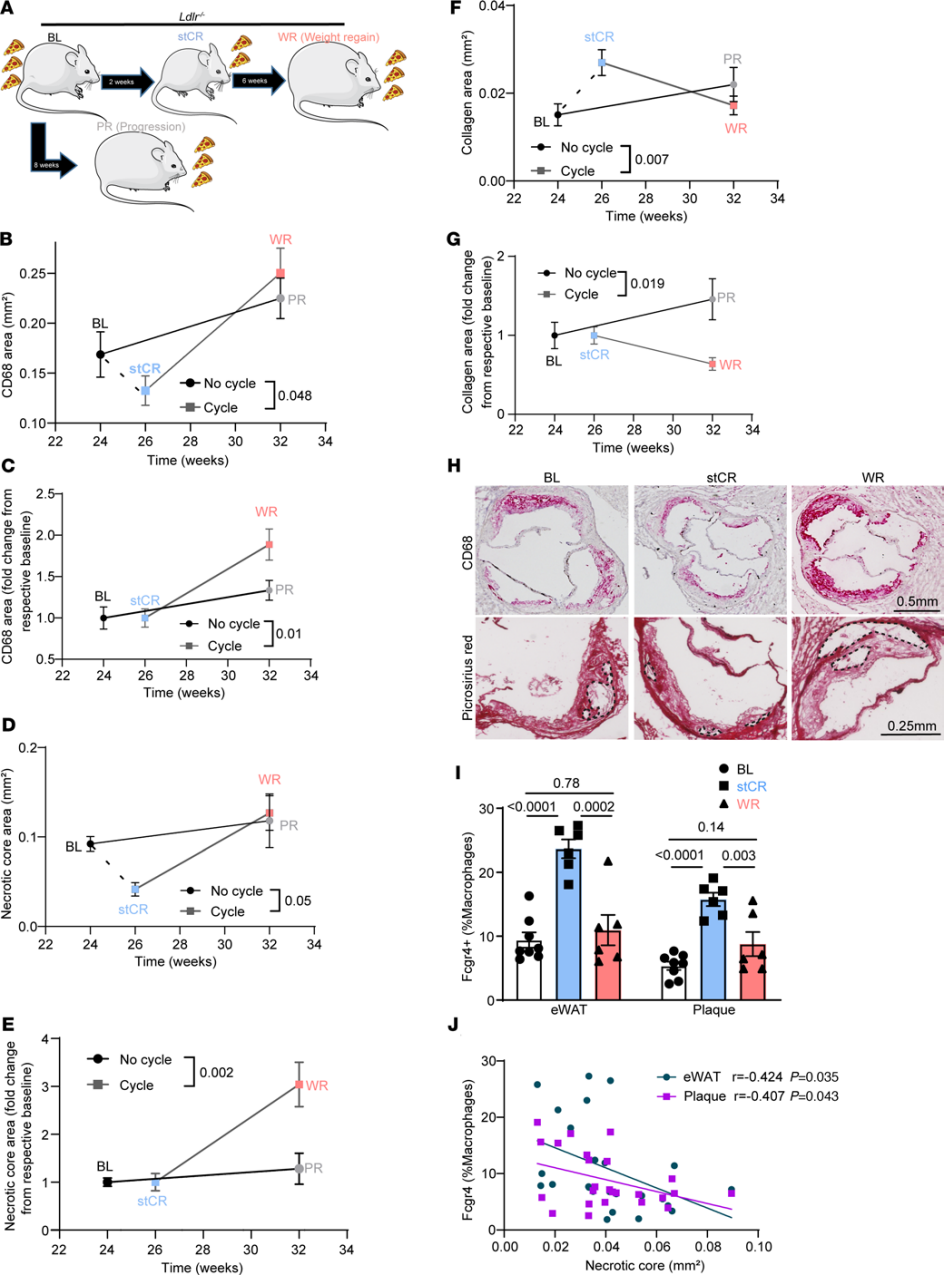
WR reverts *Fcgr4*^+^ macrophage levels to obese proportions and accelerates atherosclerosis progression. (**A**) Schematic of WR experiment. WR was induced by allowing ad libitum access to HFHC diet after a 2-week weight loss period achieved by stCR. Mice in the PR group were allowed to continue to eat ad libitum after the BL time point. (**B**–**G**) Rates of change in plaque (**B** and **C**) macrophages, (**D** and **E**) necrotic core, and (**F** and **G**) collagen areas in weight cycling versus non–weight cycling. Data are expressed as (**B**, **D**, and **F**) absolute area and (**C**, **E**, and **G**) change from respective BL (BL for PR and stCR for WR) (*n* = 11–15). (**H**) Representative aortic root images. Scale bars: 0.5 mm (top), 0.25 mm (bottom). (**I**) Flow cytometry analysis of FCGR4^+^ macrophages in eWAT and plaques (*n* = 6). (**J**) Simple linear regression showing correlation between *Fcgr4*^+^ macrophages from eWAT and plaques with plaque necrotic core. *P* values were determined via (**B**–**G** and **J**) simple linear regression and (**I**) 1-way ANOVA with Tukey’s multiple-comparison test. Data are shown as the mean ± SEM.

To investigate the mechanisms underlying increased CVD with weight fluctuation, we extended the stCR studies to include an additional WR group. For this, *Ldlr^–/–^* mice were fed a HFHC diet to establish atherosclerosis and obesity. After 24 weeks, mice were randomized to 4 groups with similar average weights with, once again, the diet composition remaining the same: (a) BL, which was harvested at this time; (b) progression (PR), which was maintained on ad libitum feeding and harvested after an additional 8 weeks; (c) stCR, which was fed 30% fewer calories daily for 2 weeks and then harvested; and (d) WR, which was put on the stCR regimen identical to group 3 and allowed free access to food thereafter for an additional 6 weeks (Figure 5A), by which time their weight gain exceeded the BL value (Supplemental Figure 5A). Weights did not significantly differ between the PR and WR groups (Supplemental Figure 5B). These feeding regimens did not significantly change plasma cholesterol levels (Supplemental Figure 5C). Additionally, WR promoted marked glucose intolerance (Supplemental Figure 5, D and E) and increased the mass of liver, eWAT, iWAT, and BAT compared with BL and stCR mice (Supplemental Figure 5, F–I).

We next examined the inflammatory state of plaques. Similar to our previous experiments (Figure 1, D and E), stCR reduced plaque macrophage content compared with BL, while WR plaques contained substantially more macrophages than in the stCR group (Supplemental Figure 5J). To investigate whether WR alters the rate of atherosclerosis progression, we compared the macrophage accumulation rate in plaques of non–weight cycling mice (i.e., from BL to PR) with those that did have weight fluctuation (from stCR to WR). As expected, macrophages gradually accumulated in plaques of mice fed a HFHC diet without weight fluctuation, resulting in an increase in their area by approximately 33% over 8 weeks. Two weeks of stCR reduced plaque macrophage area by approximately 28% compared with BL. Strikingly, WR augmented the macrophage plaque area by approximately 90% compared with the stCR group (Figure 5B). Simple linear regression analysis of plaque CD68 area showed a substantial difference in the rates of plaque macrophage accumulation between weight cycling and noncycling, indicating that WR accelerates atherosclerosis progression.

Because stCR improves several parameters in plaques, WR and PR plaques have different reference points (stCR and BL, respectively). To account for these differences, we also calculated the plaque macrophage fold change, relative to each group’s relative BL (BL for PR and stCR for WR). Notably, these analyses also showed that weight cycling results in a more rapid accumulation of macrophages in plaques (Figure 5C). Moreover, flow cytometry analysis of aortic arch macrophages revealed that compared with the stCR group, BL and WR plaque macrophages expressed more of the inflammation-associated marker Ly6C and less of the pro-resolving marker CD206 (Supplemental Figure 5K).

Further comparisons between weight cycling and noncycling showed acceleration of necrotic core formation with weight cycling (Figure 5, D and E). While there was no significant difference in macrophage or necrotic core content between BL and WR after 6 weeks of WR, the rate of their increases was accelerated in the weight cycling group. Therefore, we postulated that examining these mice at a different time point in the weight cycling protocol would show a substantial difference. To test this, we repeated the study only with the PR and WR groups and harvested the mice at 3 weeks of WR, at the time they regained all their lost weight and had comparable weights to the PR group (Supplemental Figure 5L). Importantly, at this time point we also observed a significantly higher CD68 and necrotic core content in WR plaques compared with non–weight cycled mice (Supplemental Figure 5, M and N).

Plaque collagen content, which in human plaques is thought to reflect stability (32), showed the opposite trends to macrophage and necrotic core content: Plaques from the stCR group contained significantly more collagen than in the BL or WR group; notably, the collagen gain with stCR was lost with WR (Figure 5, F and G). Representative images of plaques and necrotic cores from BL, stCR, and WR are presented in Figure 5H. Note that in addition to showing that WR accelerates atherosclerosis progression, these data recapitulate our earlier findings that stCR promotes atherosclerosis resolution.

Because *Fcgr4*^+^ macrophage accumulation in plaques and eWAT upon stCR was associated with beneficial changes in both sites, we wondered whether these cells would decrease with WR. To test this, single-cell suspensions were obtained from aortic arches and eWAT and analyzed by flow cytometry for macrophage FCGR4 levels. The results showed, again, that following stCR there were more *Fcgr4^+^* macrophages in both plaques and eWAT; however, *Fcgr4^+^* macrophage abundances in both sites reverted to their obese, BL, proportions post-WR (Figure 5I).

To further characterize the relationship between disease severity and the abundance of FCGR4^+^ macrophages, we investigated whether there was a correlation between the amount of FCGR4^+^ macrophages in eWAT or plaques with the content of plaque macrophages or necrotic core. The data show significant inverse correlations between the amount FCGR4^+^ macrophages in either tissue and plaque necrotic core size (Figure 5J). A similar correlation was seen (*P* = 0.053) with plaque macrophage content (Supplemental Figure 5O).

Taken together, the results show that WR accelerates atherosclerosis progression, with the plaques displaying rapid expansions of inflammatory cells and necrotic cores, as well as reduced collagen contents, compared with non–weight cycling conditions. Importantly, FCGR4^+^ macrophage content in either plaques or eWAT inversely correlated with disease severity in WR.

### Reprogramming of hematopoietic progenitors by WR has durable adverse effects on atherosclerosis.

**Figure 6:**
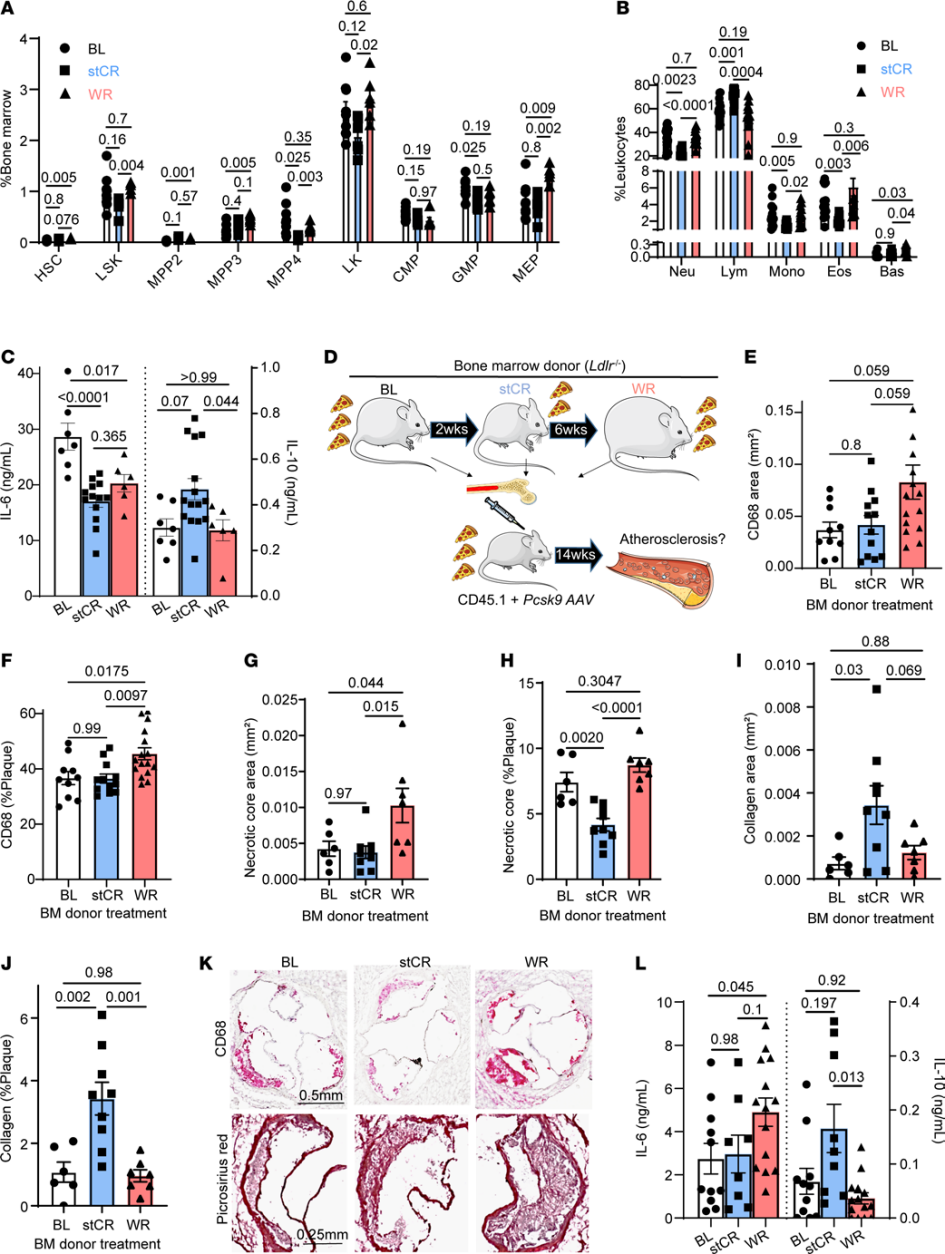
WR induces long-term pro-atherogenic reprogramming of hematopoietic progenitors. (**A**) Frequencies of bone marrow hematopoietic stem and progenitor cells (*n* = 6–9) and (**B**) circulating white blood cells (*n* = 13–17) in weight loss and regain mice (from Figure 5). (**C**) Cytokines produced by bone marrow cells treated ex vivo with LPS for 16 hours. (**D**) Schematic of bone marrow transplantation experiment (*n* = 10–14). (**E**) Plaque macrophage content expressed as total area and (**F**) percent of plaque area assessed by CD68 staining in aortic roots after 14 weeks of HFHC diet. (**G** and **H**) Plaque necrotic core and (**I** and **J**) collagen quantifications in root sections, with (**K**) representative images. Scale bars: 0.5 mm (top), 0.25 mm (bottom). (**L**) Cytokines secreted by bone marrow cells isolated from bone marrow recipient mice and treated ex vivo with LPS for 16 hours. *P* values were determined via (**A** and **B**) 2-way ANOVA and (**C**–**L**) 1-way ANOVA with Tukey’s multiple-comparison test. Data are shown as the mean ± SEM.

The data thus far show that stCR reduces, while WR hastens, atherosclerosis. We previously showed that obese eWAT promotes the expansion in bone marrow of immune progenitors with inflammatory potential, resulting in stimulation of myelopoiesis (4). Thus, we hypothesized that weight loss and regain would also influence the production and inflammatory characteristics of immune cells at the level of the bone marrow. To investigate this, the frequencies of bone marrow progenitors (including hematopoietic stem cells [HSCs], Lin-Sca-1^+^cKit^+^ [LSK], multipotent progenitors, Lin-cKit^+^ [LK], common myeloid progenitors, granulocyte-monocyte progenitors, and megakaryocyte-erythrocyte progenitors [MEPs]) and mature circulating leukocytes in the 3 conditions were determined. Several progenitor populations, including HSCs and MEPs, were most abundant in the WR group, while the stCR group had the fewest LK and LSKs (Figure 6A). This translated in the circulation to lower myeloid (neutrophils, monocytes, eosinophils, and basophils) but higher lymphoid cells in the stCR group compared with both BL and WR (Figure 6B).

Since there was a substantial decrease in leukocytes with stCR, we investigated whether this change can be driven by eWAT. Hence, mice from the adipose transplant studies presented above (Figure 4A) were analyzed for bone marrow progenitors and mature circulating immune cells. The results showed that at 2 weeks post-eWAT transplant, there were fewer circulating leukocytes, mainly lymphocytes, in the recipients of the stCR, compared with obese, eWAT (Supplemental Figure 6A). This leukocyte decrease was accompanied by reductions in several hematopoietic progenitor populations in the bone marrow (Supplemental Figure 6B). These data suggest that changes in the eWAT following stCR decrease the inflammatory effects of obese adipose tissue on immune progenitors, which would be expected to contribute to beneficial changes in atherosclerotic plaques (Figure 4, C–E).

Because eWAT from mice undergoing stCR influenced circulating immune cells and their bone marrow progenitors (Supplemental Figure 6, A and B), we hypothesized that WR induced innate immune memory–like changes (also known as trained immunity) (50) in myeloid cells and their precursors that contributed to the deleterious effects on atherosclerosis. To test this, the responses of bone marrow cells from BL, stCR, and WR mice to an inflammatory stimulus (LPS) ex vivo were examined. Supernatants were assayed 16 hours posttreatment for cytokines classically associated with inflammation (IL-6) and its resolution (IL-10). The results showed that cells from the stCR group produced the lowest amount of IL-6 and the highest amount of IL-10 compared with cells from BL and WR mice (Figure 6C). The WR cells also produced less IL-6 in response to LPS compared with cells from BL, but more than stCR cells.

To assess whether these alterations in immune responses are influenced specifically by the eWAT, we performed similar ex vivo analyses from bone marrow cells obtained from the fat transplantation study (Figure 4A). The data showed no difference in the production of IL-6, while IL-10 levels trended higher in the group that was transplanted with eWAT from stCR mice (Supplemental Figure 6C). These findings indicate that the stCR eWAT directly affects the inflammatory status of immune cells and possibly their precursors in the bone marrow by enhancing their production of pro-resolving factors.

Next, we addressed whether these quantitative and qualitative changes to immune cells and their progenitors persist long term in the context of atherosclerosis and influence plaque properties. To accomplish this, we performed bone marrow transplantation by transferring cells from the BL, stCR, or WR group (same mice as in Figure 5; all CD45.2) to naive, CD45.1 recipients. After recovery from the transplantation, we confirmed substantial bone marrow chimerism with flow cytometry using the CD45.1-CD45.2 mismatch (Supplemental Figure 6D). Mice were injected with *Pcsk9*-AAV.8 to induce LDLr deficiency (25, 43) and began HFHC diet feeding to promote atherosclerosis (Figure 6D).

After 14 weeks, all bone marrow recipient mice showed similar glucose tolerance, as measured by glucose tolerance test (AUC of 29,553 ± 2,323 in BL, 27,995 ± 1,728 in stCR, and 27,362 ± 2,175 in WR bone marrow recipients). Plasma cholesterol levels were also similar across all groups (Supplemental Figure 6E). Aortic root tissue sections show that, despite no significant differences in plaque size across recipient groups (Supplemental Figure 6F), in recipients of the WR bone marrow, compared with BL and stCR recipients, plaques had more macrophages (Figure 6, E and F) and larger necrotic cores (Figure 6G). Although the plaques in the recipients of BL and stCR bone marrow had similar size, macrophage content, and necrotic core area, the proportion of necrotic core in plaques was smallest in the stCR recipients (Figure 6H), which also had the most collagen (as either the absolute area or the percent area of the plaque that was positive; Figure 6, I and J) compared with both BL and WR. Representative images of plaques are presented in Figure 6K. Flow cytometry analysis of aortic arches showed no differences in the proportion of *Fcgr4*^+^ macrophages between the 3 groups (Supplemental Figure 6G), indicating that bone marrow progenitors do not retain long-term memory to produce *Fcgr4*^+^ macrophages upon cell transfer. These results suggest that either transient signals during stCR-induced weight loss promoted their appearance or that key epigenetic changes in the precursors were not durable, as has been shown in other situations (15, 16).

To verify that the observed changes between weight cycling and non–weight cycling bone marrows are not due to age differences of donors, we repeated the bone marrow transplant study with age-matched PR and WR donors and induced atherosclerosis in naive CD45.1 recipients with *Pcsk9*-AAV.8 (as above). Results of this experiment recapitulated our previous bone marrow transplant experiment: Recipients of bone marrow from WR donors showed several features of exacerbated atherosclerosis, including larger plaques with more macrophages and larger necrotic cores (Supplemental Figure 6, H–M).

Examination of bone marrow immune progenitors (Supplemental Figure 6N) and circulating leukocytes (Supplemental Figure 6O) of the study described in Figure 6D showed no differences in any progenitor or mature cell population across the groups. Although there were no quantitative differences in the number of circulating immune cells and progenitors, we found that bone marrow cells from WR recipients had increased IL-6 production in response to LPS ex vivo (Figure 6L). Additionally, the stCR bone marrow retained its ability to produce more IL-10 (Figure 6L), indicating that hematopoietic progenitors preserve some anti-atherogenic capabilities following stCR, mainly influencing plaque collagen content at 14 weeks of HFHC diet feeding. Taken together, the results suggest that both stCR and WR induce changes in myeloid cells and their precursors that have been seen in other settings (15, 16, 51), particularly the ability to transfer disease-associated phenotypes by bone marrow transplantation.

## Discussion

Obesity increases the risk of atherosclerosis-related CVD (52). Observational studies show that sustained weight loss decreases CVD risk, while weight cycling increases it (e.g., 5, 6, 11). To isolate the effects of weight cycling on atherosclerosis and to gain insights into underlying mechanisms, we devised a mouse model that allows weight fluctuations without confounding effects from changes in plasma cholesterol or diet composition (13). Notable results using the stCR protocol include finding (a) induction of many features of inflammation resolution in atherosclerotic plaques; (b) enrichment in both plaques and eWAT of a macrophage subpopulation distinguished by high expression of *Fcgr4*; and (c) loss of these features when mice were allowed to regain weight after stCR, likely through reprogramming of myeloid progenitors in the bone marrow.

Most studies of weight cycling have focused on the effects on systemic metabolism (e.g., glucose intolerance and insulin resistance) and include investigations of adipose tissue (53–55) and the liver (56–58). In this study, we investigated concordant changes in eWAT and atherosclerosis with weight fluctuation. Going forward, this mouse model will be a valuable tool to identify further immunological mechanisms that influence atherosclerosis and possibly other obesity-related comorbidities in the context of weight loss and regain, which is seen in >60% of patients who have dieted (48, 49). Already with a mouse model in which food intake, but not diet composition, is manipulated, we have shown profound short- and long-term changes to the immune compartment and atherosclerosis. The adherence to the same diet composition, with moderate reduction in food intake, is an important distinction of our study compared with others, which involve rounds of diet switching between high-fat and normal chow diets (e.g., 16, 55–60).

Though some diets were shown to promote epigenetic changes and inflammatory reprogramming in immune precursors (e.g., 15, 16), it is inherently difficult, if not impossible, to discriminate between the effects of the caloric content, the components of the diets, and plasma lipid levels on the metabolic or immunologic state of the mouse. For instance, Christ et al. showed that Western diet feeding of *Ldlr^–/–^* mice results in inflammatory reprogramming of immune progenitors, even at 4 weeks after switching to a chow diet that did not result in significant weight loss (15). Recently, Caslin et al. demonstrated that ATMs derived from HFD-fed mice are more inflammatory than from lean ones, a phenotype that was retained after 6 weeks of normal chow-induced weight loss (53). Yet another example of the confounding effects of changing diet composition and caloric intake at the same time is illustrated by a recent study of a preclinical model of macular degeneration, with the exacerbation of disease initially attributed to obesity. In subsequent elegant studies, the causative agent in the diet was identified as stearic acid, suggesting that it was the lower content of this fatty acid in the chow-fed mice, and not their weight loss, that protected them from macular degeneration (16).

In addition to these considerations, there is a fine line separating CR and undernutrition. While the former to a degree is well known to improve health in multiple species, the latter has been shown to promote immune dysfunction (61, 62). In the first week of diet switch from HFD to normal chow, there is a drastic decrease in food consumption, accounting for approximately 70% and approximately 35% reduction in caloric intake compared with HFD and normal chow-fed mice, respectively (55). This dramatic reduction in caloric intake may induce physiological responses that are similar to starvation or malnutrition, rather than the protective responses attributed to CR. Taken together, the points raised here support the importance in future studies of carefully dissecting the differences between various diet regimens to find the most beneficial interventions and their underlying mechanisms.

Our results reveal long-term changes to immune progenitors upon weight loss and regain (Figure 6). As noted above, recent data have indicated that obesity and intermittent Western diet feeding promote inflammatory reprogramming of macrophages (53) and immune progenitors, as well as enhancement of cardiometabolic disease (16, 53, 59, 60). ATMs from previously obese and weight cycling mice (in a model of diet switching from HFD to normal chow) were shown to retain the hyperinflamed state in response to ex vivo stimulations with toll-like receptor ligands (53). Our study is in agreement with the weight cycling, but not the weight loss results. We have shown that though the WR bone marrow cells produced more IL-6 in response to LPS than those from stCR mice, both sources of cells produced less than from obese-derived cells (Figure 6C). These inconsistencies between studies might be due to the diet regimens, as indicated above, or the different times used as study endpoints. Nonetheless, our data show that WR bone marrow produces elevated IL-6 levels even 18 weeks after bone marrow transplantation (Figure 6L), emphasizing long-term inflammatory effects.

Interestingly, the in vitro and in vivo data also demonstrate that stCR promotes long-term beneficial changes to immune progenitors. Ex vivo, bone marrow cells from stCR mice produced more IL-10 (Figure 6C), even 18 weeks after bone marrow transplant (Figure 6L). In vivo, we observed beneficial changes to plaques, mainly reduced necrotic cores and increased collagen content (Figure 6, H–J), in recipients of stCR bone marrows. This suggests that while weight loss may not necessarily overturn the inflammatory programming of bone marrow progenitors from obesity, it may induce pro-resolving features. These results further highlight the importance of retaining weight loss, since WR induced the most deleterious changes to immune progenitors (Figure 6, E–K, and Supplemental Figure 6, H–M).

Our data also indicate that the benefits of stCR on atherosclerosis may be attributed, at least in part, to *Fcgr4*^+^ macrophages. We showed that these cells accumulate in both eWAT and plaques upon stCR, but they also disappear with WR. Interestingly, the fat transplant studies (Figure 4) showed evidence of trafficking of *Fcgr4*^+^ macrophages to the plaque, suggesting this may be yet another mode of interorgan communication between adipose and other tissues (e.g., 63). The present results further suggest that Fcgr4 at the mRNA and protein levels is not only a marker of macrophages that accumulate with weight loss, but it also has functional importance. The expression of *Fcgr4* in eWAT macrophages is inversely associated with plaque necrotic cores, as shown in Figure 4, I–L, with local knockdown of *Fcgr4* in eWAT macrophages impairing the improvements in necrotic cores seen with stCR.

The data further show that overexpression of the human homolog of *Fcgr4* in macrophages enhances their efferocytotic capability (Figure 3, C and D). It is still unknown, however, how FCGR4 regulates efferocytosis beyond it being a receptor that promotes the phagocytosis of immune complexes (64). It is possible, for example, that the expression of *Fcgr4* induces a transcriptional program that facilitates other aspects of efferocytosis. Another possibility is that FCGR4 itself acts as the receptor through which dead cells are being engulfed. If the latter is correct, this would mean that antibody-mediated efferocytosis and macrophage–B cell crosstalk is necessary for this process through antibody production in B cells and engulfment of opsonized particles via FCGR4 in macrophages. Coating of apoptotic cells by antibodies and their clearance via Fc-receptors have been shown in other situations (65), and we hypothesize this may be a contributing mechanism as well. Future studies are needed to elucidate these issues. Independent of the possibilities, however, the present data suggest that the basal, or an even lower, level of *Fcgr4* expression is a limiting factor in macrophage efferocytotic activity.

In summary, we have developed a preclinical model of weight cycling that avoids confounding effects of concurrent changes in caloric intake and diet composition and that recapitulates critical cardiovascular features reported in human studies. In this model, weight loss induced the appearance of *Fcgr4*^+^ macrophages in both eWAT and plaques, which helped clear plaque necrotic cores likely through their enhanced efferocytosis capabilities. In contrast, cardiovascular benefits observed with stCR were lost with WR, with the accelerated atherogenesis attributable to durable inflammatory reprogramming of immune progenitors. This acceleration could also be viewed as the plaque “catching up” after it regressed to the state achieved by continuous progression. Going forward, the data suggest that *Fcgr4*^+^ macrophages, as well as innate immune memory, are areas in which to base future interventions to promote the benefits of weight loss and prevent the deleterious effects of WR on metabolic inflammation, currently major clinical issues in CVD prevention.

## Methods

### Sex as a biological variable.

Eight- to twelve-week-old male *Ldlr^–/–^*, WT C57BL/6J (CD45.2^+^), and CD45.1^+^ mice were used for all experiments, with breeding pairs originally purchased from The Jackson Laboratory.

Only male mice were used due to females’ resistance to develop obesity. Thus, further investigations are needed to determine whether there are sex-specific differences in the response to weight loss and regain.

### Statistics.

GraphPad Prism 9 (GraphPad Software) was used for statistical analysis. Data are expressed as mean ± SEM. Comparison of 2 groups was analyzed using a 2-tailed Student’s *t* test. With 3 or more groups, statistical analysis was performed using 1-way ANOVA, with Tukey’s multiple comparisons testing and Gaussian distribution. Comparison of 2 parameters for 2 or more groups was performed using a 2-way ANOVA, with Šidák’s multiple comparison testing. All data were checked for normality, and nonparametric testing was performed when data were not normally distributed. *P* < 0.05 was considered significant.

### Study approval.

All mouse procedures were approved by the IACUC of the New York University School of Medicine.

### Data availability.

Data are publicly available in GEO under GSE225077 (bulk RNA-Seq of *Fcgr4*^+^ macrophages) and GSE141036 (scRNA-Seq of eWAT and plaque leukocytes). Further information can be found in Supplemental Methods and in the Supporting Data Values file.

## Supplementary Material

Supplemental data

Supplemental table 1

Supplemental table 2

Supplemental table 3

Supplemental table 4

Supporting data values

## Version 1 07/08/2025

In-Press Preview

## Version 2 09/16/2025

Electronic publication
