# Supplementary material for: Caloric restriction promotes resolution of atherosclerosis in obese mice, while weight regain accelerates its progression
Source: J Clin Invest. 2025 Jul 8;135(18):e172198. doi: 10.1172/JCI172198 (PMC12435835; doi:10.1172/JCI172198)
Supplement: Supplemental data [file jci-135-172198-s009.pdf]

SUPPLEMENTAL METHODS

Weight cycling model

Mice were C57BL/6 and CD45.2 (an isoform of a pan-leukocyte marker) unless otherwise indicated in Results. Obesity was induced by feeding mice a high-fat –high-cholesterol (HFHC) diet (Research Diets, 17052507i; New Brunswick, NJ) *ad libitum* for 24 weeks (baseline; BL). This is a custom diet comprised of the standard 60% high-fat diet (catalog number D12492), with 0.3% added cholesterol. Depending on the experimental design (as indicated in Results), LDLr deficiency was accomplished in different ways to induce hypercholesterolemia and atherosclerosis: 1) *Ldlr-/-* mice were purchased from Jackson Laboratory and a colony established at the NYU Grossman School of Medicine; 2) Mice received weekly injections of 5mg/kg antisense oligonucleotide (ASO) targeting the LDL receptor (generously provided by Ionis Pharmaceuticals) for the duration of HFHC diet feeding; 3) Mice received injections of an AAV.8 vector expressing *Pcsk9* (purchased from the University of Pennsylvania Vector Core), as we have done before(1). The second method allowed us to lower plasma cholesterol levels by withdrawing the ASO in experiments not related to the present study. The third method allowed making any type of mouse hypercholesterolemic and was of particular use with CD45.1 mice, to avoid the delay of multiple crosses with *Ldlr-/-* mice, which are available only as CD45.2 from the Jackson Laboratory.

Weight loss was induced by restricting daily caloric intake of obese mice by 30% for 2 weeks (short-term caloric restriction, stCR), as described by Ferrante and colleagues(2). After weight loss, a group of mice was again provided with HFHC diet *ad libitum* for 3 or 6 weeks (weight regain, WR).

Glucose tolerance test

Glucose tolerance test (GTT) was performed after fasting for 6h and acclimation to the testing room with access to water. Mice were injected intraperitoneally (i.p.) with D-glucose (Crystalgen

300-341-1000) at 2g per kg of body weight. Blood glucose levels were measured via tail sampling using a glucometer (Contour Next EZ, Bayer). Data are presented as areas under the curves, with no minimum subtracted.

Lipid and lipoprotein analyses

Total cholesterol (TC) was measured using the enzymatic assay Total Cholesterol E Kit (Wako Life Science, NC9138103; Richmond, VA). Plasma lipoproteins were separated using fast-performance liquid chromatography with two Superose 6 10/300 GL columns (GE Healthcare; Boston, MA) on a Shimadzu HPLC system (Columbia, MD).

Plaque morphometrics and immunohistochemistry

Mice were euthanized at the end of each dietary regimen and blood was collected via cardiac puncture, followed by perfusion with saline at physiologic pressure. Hearts were then removed and embedded in OCT (Sakura, 4583; Torrance, CA) and immediately frozen at -80^{0}C, while arches were removed and kept in PBS for same day flow cytometry. Aortic root sections (6 µm) were stained for CD68 (Bio-Rad MCA1957; Hercules, CA) to detect macrophages.

Necrotic cores were initially identified by inspection of hematoxylin and eosin-negative acellular areas in the intima, as described previously(3). Briefly, necrotic cores were defined as areas with low or no extracellular matrix. Consecutive sections (6 µm apart) of aortic roots were stained with H&E and either an antibody for CD68 or picrosirius red (PolySciences 24901-500; Niles, IL). The picrosirius red images were used to confirm the necrotic core area selection in the CD68-stained slides, then used for quantification. The results for each mouse represent data from ~12 serial sections, spanning ~400 µm. Examples of the two types of staining (H&E plus CD68 or H&E plus picosirius red) of adjacent sections are shown in Supp. Fig. 7.

TUNEL staining was performed according to the manufacturer's instructions (Invitrogen C10619; Waltham, MA). In brief, thawed aortic root sections were fixed with 4% paraformaldehyde for 15 minutes at 37^0C and then proteinase K solution was added for 5 minutes. Slides were then washed and fixed once more with 4% paraformaldehyde for 5 minutes at 37^0C. TdT buffer was then added for 10 minutes, removed and TdT reaction solution added for 60 minutes, after which slides were washed and incubated with 0.1% TritonX-100 for 5 minutes. Slides were then added the TUNEL reaction cocktail for 30 minutes at 37^0C, washed and then proceeded to FCGR4 and CD68 staining. Antibodies for both FCGR4 (SinoBio 50036-T24; Wayne, PA) and CD68 (as above) were added at a 1:250 dilution and incubated for 1 hour at room temperature. Secondary antibodies were used at a 1:400 dilution, and added after washing with PBS. After 1 hour incubation, slides were washed again and mounted using Prolong Gold (Invitrogen P36934; Waltham, MA). Control sections stained with secondary antibodies only were used to determine background staining.

Isolation of immune cells from aortic arch and adipose for flow cytometry

Cell suspensions from plaques were generated after aortic arch (see below) and adipose digestion(4), and sorted using BD FACS Aria II SORP. To single-cell suspensions was added a live/dead cell staining (Invitrogen, L10119; Waltham, MA) and the following antibodies:BV510 anti-CD45 (103137), PE anti-CCR2 (150609), PE-Cy7 anti-Ly6G (127618), BV711 anti-CD11b (101241), BV570 anti-Ly6C (128030), BV650 anti-CD11c (117339), BV421 anti-B220 (117339), BV421 anti-CD3 (100227), AF647 anti-CD206 (141712), PE-Cy7 anti-CD14 (123316), PerCP-Cy5.5 anti-FCGR4 (149518). All antibodies were purchased from Biolegend (San Diego, CA), as well as BV421 anti-SiglecF (BD Bioscience, 562681; Franklin Lakes, NJ) and PE anti-CD163 (eBioscience, 12-1631-80; San Diego, CA). Single stained samples were used as controls.

Aortic digestion was performed by mincing the aortic arch and branches with a blade. Minced tissues were suspended in 3mL digestion buffer: Hank's Balanced Salt Solution, 1mM

EDTA, 1% BSA, 0.77mg/mL Liberase (Roche, 273582; Basel, Switzerland), 0.1mg/mL Hyaluronidase (Sigma Aldrich, 3506; St. Louis, MO) and 0.06mg/mL DNaseI (Sigma Aldrich, DN25; St. Louis, MO). Tissue was then transferred to C-tubes, placed in a gentleMACS dissociator (both from Miltenyi Biotech; Bergisch Gladbach, Germany) and digested for 15 minutes in 37^0C. Suspensions were filtered using a 100μm nylon mesh and centrifuged for 5 minutes. Cell pellets contained single cells that were next stained for flow-cytometry analysis.

RNA extraction and quantitative real-time PCR analysis

Total RNA was extracted from cells using TRIzol (Invitrogen) and Direct-zol Miniprep kit (Zymo Research) followed by cDNA synthesis using the Verso cDNA kit (Thermo Scientific). SYBR Green-based real-time qPCR was performed using the ABI PRISM 7300 sequence detection system (Applied Biosystems). Gene expression was normalized to *Hprt* expression and assessed using Δ(ΔCt). Primer sequences were as follows: *Tnfa* (Fw *5'-GATCTCAAAGACAACCAACATGTG-3', Rv 5'-CTCCAGCTGGAAGACTCCTCCCAG-3'), Nos2 (Fw 5'-CTGATGGCAGACTACAAAGACG-3', Rv TGGCGGAGAGCATTTTTGAC-3'), Il6 (Fw 5'-CTATACCACTTCACAAGTCGGA-3', Rv 5'-GAATTGCCATTGCACAACTCTTT-3'), Fcgr4 (Fw 5'-ATGTGGCAGCTACTACTACCA-3', Rv 5'-ACCCACTTGGGGTCTAGGTTC-3')*
Fcgr3a (Fw 5' GGTTTGGTCCCTTTAGGGCT -3', Rv 5'- TTGGGAGATCTTCAGTCCGC -3'), Mrc1 (Fw 5'- CTCTGTTCAGCTATTGGAGCG-3', Rv 5'-CGGAATTTCTGGGATTCAGCTTC-3'), Arg1 (Fw 5'-CTCCAAGCCAAAGTCCTTAGAG-3', Rv 5'-AGGAGCTGTCATTAGGGACATC-3')

Isolation and treatment of primary macrophages

Bone marrow derived macrophages (BMDMs) were obtained by harvesting femur and tibia, cutting the edges of each bone and spinning them for 20 seconds at 8000 rpm, in an Eppendorf tube containing 100 μL of PBS. After red blood cell lysis for 10 minutes at room temperature, cells were plated in 48 well plates (40 wells per mice) in DMEM (1gL/glucose) containing 10%

FBS, 1% penicillin/streptomycin and 10ng/mL M-CSF (Biolegend, 576406; San Diego, CA). Fresh medium was added on day 3. On day 7, macrophages were treated for overexpression of *Fcgr3a* (see below).

Cells were treated with either 10ng/mL LPS (Sigma Aldrich, L4391; St. Louis, MO) or IL-4 (Peprotech, 214-14; Cranbury, NJ) for 6 hours, or re-seeded in chamber slides (500,000 cells per well) and incubated overnight for the efferocytosis assay (see below) on the following day.

Total bone marrow cells were obtained by flushing bone marrow from femurs and tibias and plating on 96-well flat-bottom well culture plates (270,000 cells/well) in complete DMEM, as above. After a 2-hour resting period in the incubator (37^0C, 5% CO_2), cells were stimulated for 16 hours with 100ng/mL LPS, or media only (as control). Subsequently, supernatants were collected for cytokines measurement through ELISA.

mRNA delivery and overexpression in macrophages

mRNA-liposome complexes were prepared using Lipofectamine™ MessengerMAX™ Transfection reagent (Thermo Scientific, LMRNA001). In brief, for each transfection reaction (1 well of a 12 well plate), 1 µg of either scrambled or *Fcgr3a* mRNA (m6A-modified) were diluted in 50 µL of Opti-MEM™ and gently mixed. Separately, 1.5 µL of Lipofectamine™ MessengerMAX™ reagent was diluted in 50 µL of Opti-MEM™ and incubated for 5 minutes at room temperature. The diluted mRNA was then added to the diluted Lipofectamine™ MessengerMAX™ reagent and incubated for 15 minutes at room temperature to allow complex formation.

Differentiated macrophages were washed with PBS and cultured in DMEM without antibiotics for the transfection procedure. The mRNA-liposome complexes were added dropwise to the macrophages, and the plates were gently swirled to ensure even distribution of the complexes. The cells were then incubated at 37°C for 6 hours, after which the transfection medium was replaced with fresh DMEM medium containing 10% FBS and antibiotics. The

macrophages were incubated for an additional 30 hours to allow for mRNA translation, after which the cells were washed and serum free DMEM media was added with LPS or IL-4 (as above).

Efferocytosis

Macrophages were irradiated with ultraviolet light for 30 minutes to induce apoptosis, followed by labeling with CellTracker™ orange dye (Invitrogen, C34551), for 30 min. Cells were washed once with 10 mL of serum free media and suspended in RPMI+ 10% FBS and 1% penicillin/streptomycin and kept in the incubator for 3 hours. After that, apoptotic cells were applied to effector macrophages (2.5:1 apoptotic:effector) . After 40 min incubation, media were aspirated and effector macrophages were washed three times with PBS, followed by fixation with formalin for 15 min. Efferocytotic events were calculated as unstained cells (i.e., effector macrophages) with red label inside. Results were expressed as % of total effector macrophages that were positive for the red stain.

Bone marrow progenitor isolation and quantification

Bone marrow cells were obtained from femur and tibia (same as for obtaining BMDMs, as described above). After the red blood cell lysis, cells were counted and added a green live/dead stain (Invitrogen, L34969; Waltham, MA) in PBS and kept at 4ºC, in the dark, for 30 minutes. Cells were then spun, supernatants discarded, and to the cells was added a cocktail of antibodies: from eBioscience (San Diego, CA)- FITC anti-GR1 (11-5931-82), FITC anti-CD3 (11-0031-82), FITC anti-CD4 (11-0041-82), FITC anti-CD8 (11-0081-82), FITC anti-Ter119 (11-5921-82), FITC anti-CD19 (11-0193-82), FITC anti-NK1.1 (11-5941-82), FITC anf-CD2 (11-0021-82), APC anti-CD34 (50-0341-82), and from Biolegend (San Diego, CA)- FITC anti-CD11b (101206), PE-Cy7 anti-Sca1 (108114), APC-Cy7 anti-cKit (105826), PE anti-CD135 (135306), PerCP-Cy5.5 anti-CD150 (115922), BV605 anti-CD48 (103441) and BV711 anti-CD16/32 (101337). Cells were kept at 4ºC,

in the dark, overnight, after which they were washed and analyzed using an LSRII (BD Biosciences; Franklin Lakes, NJ).

Adipose tissue transplantation

CD45.1 adipose tissue donors were fed a HFHC diet for 24 weeks, after which some mice were calorically restricted for 2 weeks (given 70% of their daily food consumption, as above). Recipient mice were lean male *Ldlr*$^{-/-}$ with established atherosclerosis, achieved by low-fat high-cholesterol diet feeding (Research Diets)(5) for 20 weeks. The use of a low-fat diet prevented the potentially confounding development of obesity. The transplantation studies were conducted following standard antiseptic surgical techniques. eWAT from either obese or stCR CD45.1 mice was immediately excised post-euthanasia, washed in sterile PBS, and 400mg were transplanted into the subcutaneous anterior dorsal region of recipient mice, as previously described(6). Two days pre-transplant, recipients were switched to a low-cholesterol diet (chow), to gradually lower plasma cholesterol and allow reparative signals (if any) from the transplanted adipose tissue to influence plaques or bone marrow. Tissues of eWAT recipients were harvested 2 weeks post-transplant.

Murine white blood cell (WBC) counts

Total WBC counts in freshly isolated blood were performed by collecting tail blood in EDTA-contained tubes and analyzed using a hematology cell counter (Heska Element HT-5).

***Fcgr4* siRNA particles and treatments in vivo**

Glucan-shell, PEGylated particles containing scrambled (Dharmacon D-001810-04; Lafayette, CO) or *Fcgr4* (Dharmacon J-053664-17) siRNA were generated as previously described(7-9). WT males were injected with *Pcsk9* AAV.8TBGmPCSK9D377Y (2×10^{12} viral particles/mouse, Penn Vector Core; Philadelphia, PA)(10), and placed on a HFHC diet to induce LDLr-deficiency and

atherosclerosis. After 20 weeks the obese mice were randomized to groups with similar body weight and stCR was started. At the same time mice were injected with 200uL particles i.p. once a day for 14 days.

Bone marrow (BM) transplantation

BM cells were harvested from femora and tibias of *Ldlr*$^{-/-}$ donor mice (BL, CR, WR) and cryopreserved. On the day of transplantation, 1x10^6 thawed cells were suspended in 0.2 mL PBS and injected retro-orbitally into male CD45.1 recipients that were lethally x-irradiated with a total dose of 10Gy. After 4 weeks of recovery, recipient mice were injected i.p. with the *Pcsk9* AAV8 (as above). Animals were provided with HFHC diet for 14 weeks and harvested thereafter.

Cytokine ELISA

Purified and biotinylated IL-6 (504502 and 504601, respectively) and IL-10 (505002 and 504906, respectively) antibodies were purchased from Biolegend (San Diego, CA). Purified antibodies were used to coat an ELISA plate, according to the manufacturer's instructions, and incubated overnight in 4^0C. The plate was then washed 4 times in PBS containing 0.05% tween (PBST) and blocked with 1% BSA in PBS. After 1 hour incubation at room temperature, medium conditioned by bone marrow cells was added and incubated in 4^0C overnight. The plate was then washed 4 times in PBST and added was one of the biotinylated antibodies, according to the manufacturer's instructions, and incubated for 1 hour at room temperature. The plate was then washed 4 times in PBST and next added was alkaline phosphatase conjugated to streptavidin, from Jackson ImmunoResearch (016050084; West Grove, PA) according to the manufacturer's instructions. After 30 minutes incubation at room temperature the plate was washed 4 times with PBST and alkaline phosphatase substrate was added (Sigma Aldrich, S0942; St. Louis, MO). The plate was analyzed using a plate reader, for emission in 405nm. Standard curves were established using

mouse recombinant IL-6 (Peprotech, 216-16; Cranbury, NJ) and IL-10 (Biolegend, 575804; San Diego, CA).

Methods for scRNAseq analysis

Read alignment, barcode de-convolution, and UMI (unique molecular identifier) counting

Following sequencing, we used the CellRanger Single Cell Software Suite v 3.1.0 to de-multiplex individual cells, process UMIs, and count UMIs per gene, following the standard pipeline and default parameters. Briefly, by using *cellranger count*, FASTQ files were generated and aligned to the mouse mm10 genome, sequencing reads were filtered by quality score, and cell barcodes and UMIs were assigned to each read. The filtered gene expression matrices from the plaque BL and stCR samples were then used for downstream analyses.

Filtering cells

To identify low-quality cells and doublets, we visualized the distribution of the number of UMI detected for each cell, the number of genes expressed, and the percentage of reads that were coming from mitochondrial genes using Seurat v 4.1.1(11). To remove potential doublets, i.e. multiple cells captured in a single gel bead emulsion, we removed cells that were outliers for the number of expressed genes, using a cutoff of 6500 genes for plaque BL, 4500 genes for eWAT BL, 3000 genes for plaque stCR, and 4000 genes for eWAT stCR. We also removed cells that had > 7.5% of their reads aligned to mitochondrial genes.

Normalization and alignment of datasets

To merge our plaque datasets with those from Weinstock et al.(4), we first normalized each of the 4 samples (Plaque BL and CR, and Fat BL and CR) using the *SCTransform* within Seurat. We then integrated the datasets using *SelectIntegrationFeatures* with default parameters and *nfeatures =3000*. We then ran *PrepSCTIntegration*, *FindIntegrationAnchors* and *IntegrateData* using default parameters, but integrating all genes in the dataset, not just the integration features.

Dimensionality reduction, cluster finding, and cell type annotation

After integrating the 4 datasets, we ran the following functions on the "integrated" assay using Seurat: *RunPCA*, *RunUMAP*, *FindNeighbors*, and *FindClusters*, using 30 PCAs for *RunUMAP* and *FindNeighbors*. We then annotated the clusters with SingleR 1.4.1, using the Zernecke et al. meta-analysis(12) as a reference dataset. A permutation test followed by FDR correction for multiple testing in the R package scProportionTest(13)was used to identify significant differences (FDR < 0.05) in cell type abundance between stCR and BL in each tissue.

Downsampling and differential expression

The plaque BL sample had significantly deeper sequencing coverage than the other 3 samples, as shown in Supp. Fig. 2A. This resulted in a skew towards genes that had higher expression in plaque BL when we ran differential expression analyses. To correct this likely source of bias, we downsampled all 4 samples to include a maximum of 10,000 UMI per cell using the *SampleUMI* function within Seurat. We retained the same cluster identities as in the full dataset, and ran differential expression using the downsampled data to get more symmetrical numbers of differentially expressed genes between Plaque BL and the other samples. Differential gene expression was assessed using a Wilcoxon test in Seurat. P-values were adjusted for multiple testing using Bonferroni correction. Genes were selected as differentially expressed in eWAT using adjusted p-value < 0.1. Genes were selected as differentially expressed in the plaque using adjusted p-value < 0.1 and requiring at least a 2-fold change in expression.

Cell-cell communication

Metadata information on cluster membership and raw counts were extracted for each sample and normalized as counts per 10,000; these were then used to run the *cellphonedb method statistical_analysis(14)* for each individual sample for the analysis. The *significant_means* output was then parsed using custom scripts to plot the total number of significant interactions per cluster pair.

Bulk RNA sequencing analysis

Samples from the bulk sequencing analysis were first mapped to mm10 using STAR v 2.6.0, and gene counts were generated using the FeatureCounts function within subread v 1.6.3. We then used DESeq2 v 1.30.1 to detect differential expression between the FCGR4 positive and FCGR4 negative macrophages, using a paired sample approach with the model *design = ~mouse + treatment* where mouse was the individual mouse and treatment was either Fcgr4 positive or negative. We used the Bioconductor package clusterProfiler v 3.18.1 to perform GO and KEGG enrichment on genes significantly differentially expressed between Fcgr4 positive and negative macrophages.

Statistical analysis

GraphPad Prism 9 (GraphPad Software) was used for statistical analysis. Data are expressed as mean ± SEM. Comparison of two groups was analyzed using a 2-tailed student t-test. With three or more groups, statistical analysis was performed using one-way ANOVA, with Tukey's multiple comparisons testing and Gaussian distribution. Comparison of two parameters for two or more groups was performed using a two-way ANOVA, with Sidak's multiple comparison testing. All data were checked for normality and non-parametric testing was performed when data were not normally distributed. $p<0.05$ was considered significant.

REFERENCES

1. Peled M, Nishi H, Weinstock A, Barrett TJ, Zhou F, Quezada A, et al. A wild-type mouse-based model for the regression of inflammation in atherosclerosis. *PLoS One.* 2017;12(3):e0173975.
2. Kosteli A, Sugaru E, Haemmerle G, Martin JF, Lei J, Zechner R, et al. Weight loss and lipolysis promote a dynamic immune response in murine adipose tissue. *J Clin Invest.* 2010;120(10):3466-79.
3. Yurdagul A, Jr., Subramanian M, Wang X, Crown SB, Ilkayeva OR, Darville L, et al. Macrophage Metabolism of Apoptotic Cell-Derived Arginine Promotes Continual Efferocytosis and Resolution of Injury. *Cell Metab.* 2020;31(3):518-33 e10.
4. Weinstock A, Brown EJ, Garabedian ML, Pena S, Sharma M, Lafaille J, et al. Single-Cell RNA Sequencing of Visceral Adipose Tissue Leukocytes Reveals that Caloric

Restriction Following Obesity Promotes the Accumulation of a Distinct Macrophage Population with Features of Phagocytic Cells. *Immunometabolism.* 2019;1.
5. Hartvigsen K, Binder CJ, Hansen LF, Rafia A, Juliano J, Horkko S, et al. A diet-induced hypercholesterolemic murine model to study atherogenesis without obesity and metabolic syndrome. *Arterioscler Thromb Vasc Biol.* 2007;27(4):878-85.
6. Stanford KI, Middelbeek RJ, Townsend KL, An D, Nygaard EB, Hitchcox KM, et al. Brown adipose tissue regulates glucose homeostasis and insulin sensitivity. *J Clin Invest.* 2013;123(1):215-23.
7. Aouadi M, Tencerova M, Vangala P, Yawe JC, Nicoloro SM, Amano SU, et al. Gene silencing in adipose tissue macrophages regulates whole-body metabolism in obese mice. *Proc Natl Acad Sci U S A.* 2013;110(20):8278-83.
8. Barreby E, Sulen A, and Aouadi M. Glucan-Encapsulated siRNA Particles (GeRPs) for Specific Gene Silencing in Adipose Tissue Macrophages. *Methods Mol Biol.* 2019;1951:49-57.
9. Tesz GJ, Aouadi M, Prot M, Nicoloro SM, Boutet E, Amano SU, et al. Glucan particles for selective delivery of siRNA to phagocytic cells in mice. *Biochem J.* 2011;436(2):351-62.
10. Bjorklund MM, Hollensen AK, Hagensen MK, Dagnaes-Hansen F, Christoffersen C, Mikkelsen JG, et al. Induction of atherosclerosis in mice and hamsters without germline genetic engineering. *Circ Res.* 2014;114(11):1684-9.
11. Hao Y, Hao S, Andersen-Nissen E, Mauck WM, 3rd, Zheng S, Butler A, et al. Integrated analysis of multimodal single-cell data. *Cell.* 2021;184(13):3573-87 e29.
12. Zernecke A, Winkels H, Cochain C, Williams JW, Wolf D, Soehnlein O, et al. Meta-Analysis of Leukocyte Diversity in Atherosclerotic Mouse Aortas. *Circ Res.* 2020;127(3):402-26.
13. Miller SA, Policastro RA, Sriramkumar S, Lai T, Huntington TD, Ladaika CA, et al. LSD1 and Aberrant DNA Methylation Mediate Persistence of Enteroendocrine Progenitors That Support BRAF-Mutant Colorectal Cancer. *Cancer Res.* 2021;81(14):3791-805.
14. Efremova M, Vento-Tormo M, Teichmann SA, and Vento-Tormo R. CellPhoneDB: inferring cell-cell communication from combined expression of multi-subunit ligand-receptor complexes. *Nat Protoc.* 2020;15(4):1484-506.

Supplemental Figures

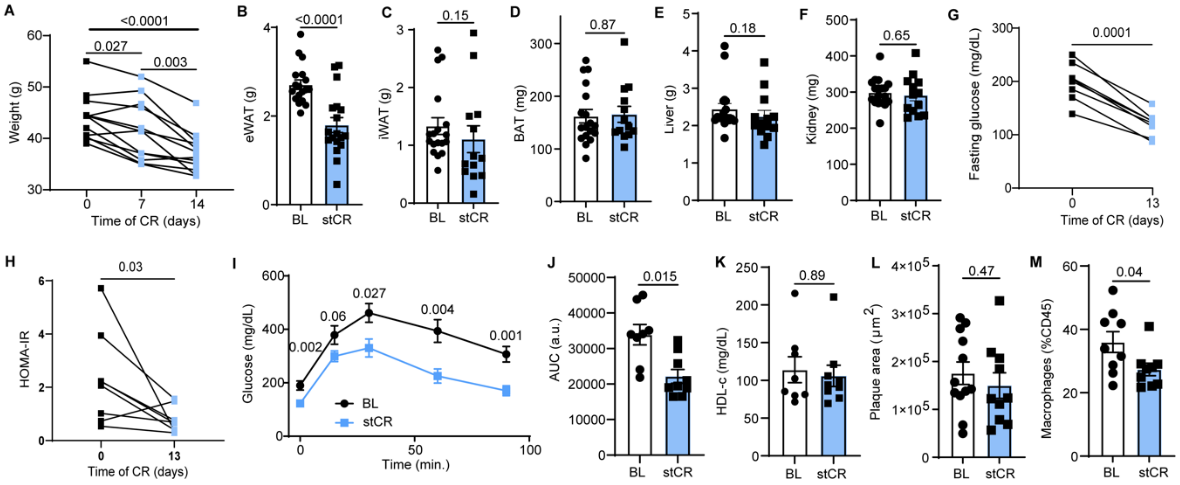

Supp. Fig. 1: stCR promotes weight loss and improves metabolic parameters.

(A) Body weight assessed during caloric restriction. (B-F) Tissue weights at the end of the experiment: (B) eWAT, (C) iWAT, (D) BAT, (E) liver (F) and kidney. (G) Fasting glucose levels at baseline and after stCR. (H) Homeostatic Model Assessment for Insulin Resistance (HOMA-IR) at baseline and after stCR. (I, J) Glucose tolerance test performed in baseline vs. caloric restricted mice. (K) HDL-C measurements. (L) Total plaque area quantified in root sections. (M) Proportion of macrophages from all white blood cells in aortic arches, measured by flow-cytometry. P-values were determined via (A) one-way ANOVA with Tukey's multiple comparisons test, (B, F-L) two-tailed Student's t-test and (C-E, M) Mann-Whitney test. Data are shown as the mean ± SEM.

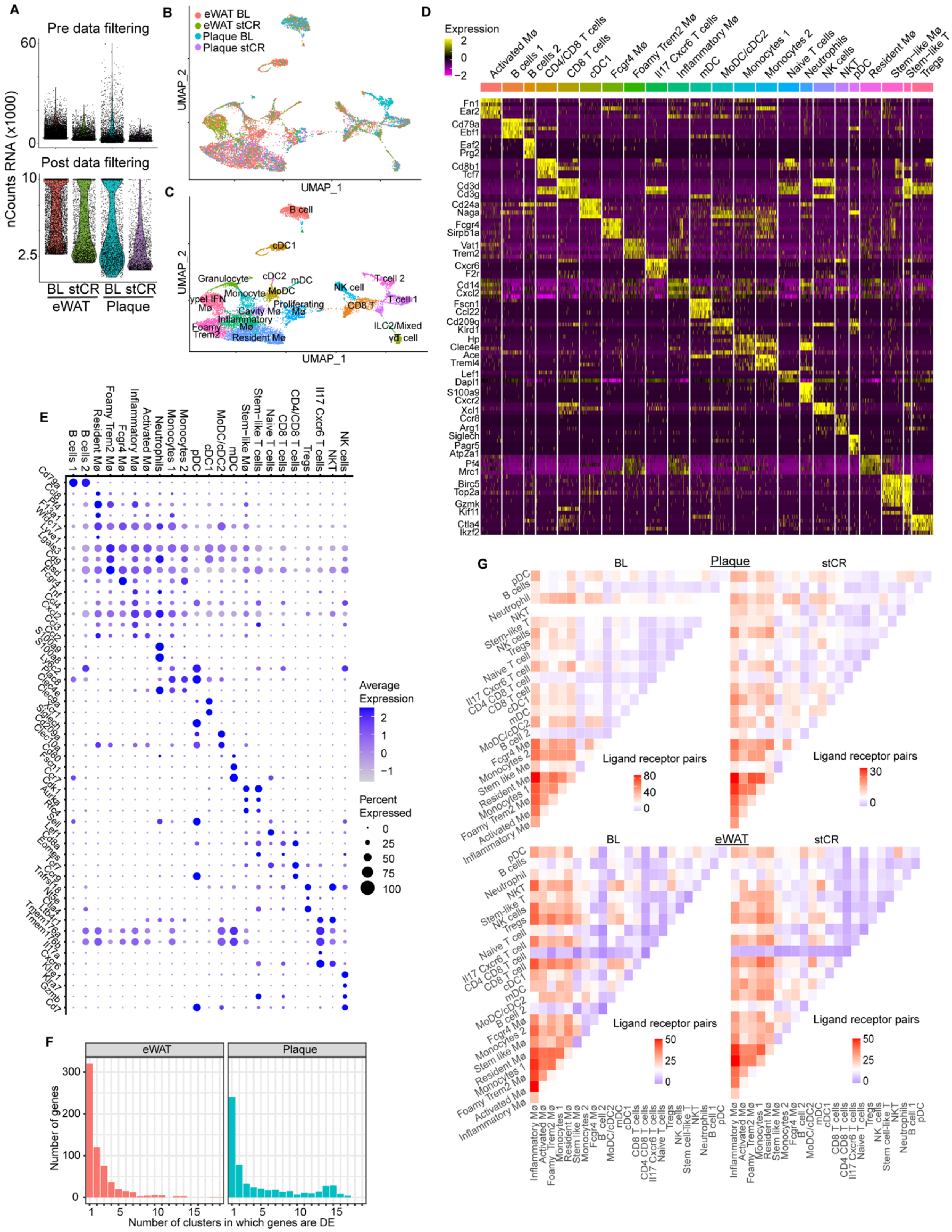

A
Pre data filtering
Post data filtering
nCounts RNA (x1000)
BL stCR BL stCR
eWAT Plaque
B
eWAT BL
eWAT stCR
Plaque BL
Plaque stCR
UMAP_1
UMAP_2
C
B cell
cDC1
Granulocyte
cDC2
mDC
MoDC
Monocyte
NK cell
T cell 2
T cell 1
CD8 T
Proliferating Mø
Cavity Mø
Inflammatory Mø
Resident Mø
ILC2/Mixed
γδ cell
D
Expression
E
Average Expression
Percent Expressed
F
eWAT
Plaque
Number of genes
Number of clusters in which genes are DE
G
Plaque
eWAT
BL
stCR
Ligand receptor pairs

Supp. Fig. 2: Single-cell RNA-seq of plaque and eWAT leukocytes in obesity and weight loss.

(A) Quality control of scRNAseq data pre- and post-filtering for cells containing >10,000 unique RNA reads (see methods). UMAPs color coded by (B) sample type and (C) closest match compared to Zernecke et al. (D) Top 5 marker genes per cluster. (E) Expression of marker genes defined by Zernecke et al., used to annotate leukocyte clusters in our dataset. (F) Number of clusters with shared DEGs (comparing stCR and BL) in eWAT (red) and plaques (blue). (G) Cell-cell interaction analysis, presenting the number of receptor-ligand pairs across all clusters in plaque (top) and eWAT (bottom) in obese (left) and stCR (right). Data are shown as the mean ± SEM.

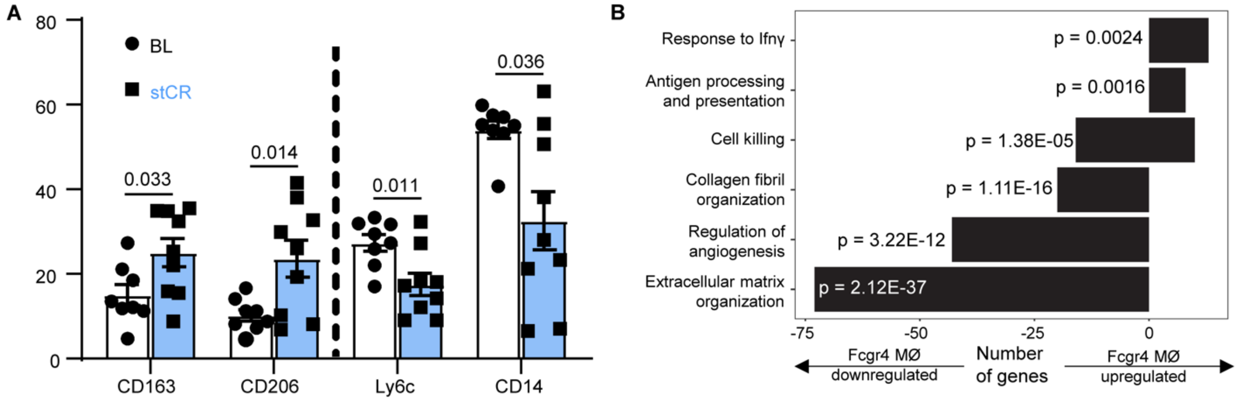

<u>Supp. Fig. 3: stCR induces a less inflammatory, pro-resolving phenotype of plaque macrophages, partly through induction of *Fcgr4*+ macrophages.</u>

(A) Flow cytometry analysis of plaque macrophages after aortic arch digestion (n=8-10). P-values were determined via two-tailed Student's t-test except for CD14, which was determined by Mann-Whitney test. (B) GO pathways increased and repressed in FCGR4+ macrophages ("Fcgr4 MΦ") from adipose tissue following caloric restriction. Enriched GO terms have adjusted p-value < 0.05. Data are shown as the mean ± SEM.

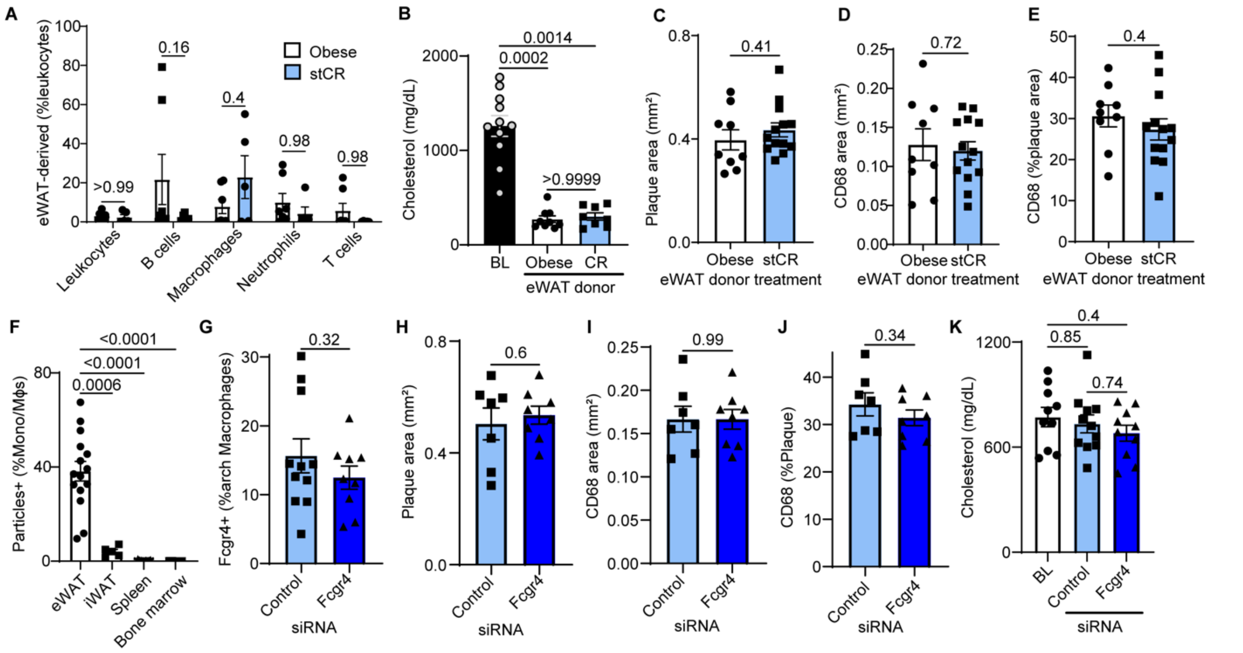

Supp Fig. 4: *Fcgr4*+ macrophages from eWAT reduce plaque necrotic core, but do not change plaque macrophage content.

(A) Flow cytometry analysis of plaque leukocytes of eWAT recipient mice, 2 weeks after transplantation (n=5-6). (B) Plasma cholesterol at tissue harvest. (C) Quantification of total plaque area and (D-E) macrophage content in aortic root sections (n=7-11). (F) Flow cytometry analysis of particle engulfment by macrophages from different tissues (n=5-12). (G) Flow cytometry analysis of FCGR4+ macrophages (Fcgr4+ on Y-axis) in aortic arch of mice receiving either control or *Fcgr4* siRNA-containing particles (n=9-12). (H) Quantification of total plaque area and (I-J) macrophage content of aortic root sections, after injections of control or *Fcgr4* siRNA particles. (K) Plasma cholesterol after 2 weeks of siRNA-carrying particle treatment. P-values were determined via (A) two-way ANOVA with Sidak (F,K) one-way ANOVA with Tukey's multiple comparisons test, (E) Kruskal-Wallis with Dunn's multiple comparisons test and (B-D, G-J) two-tailed Student's t-test. Data are shown as the mean ± SEM.

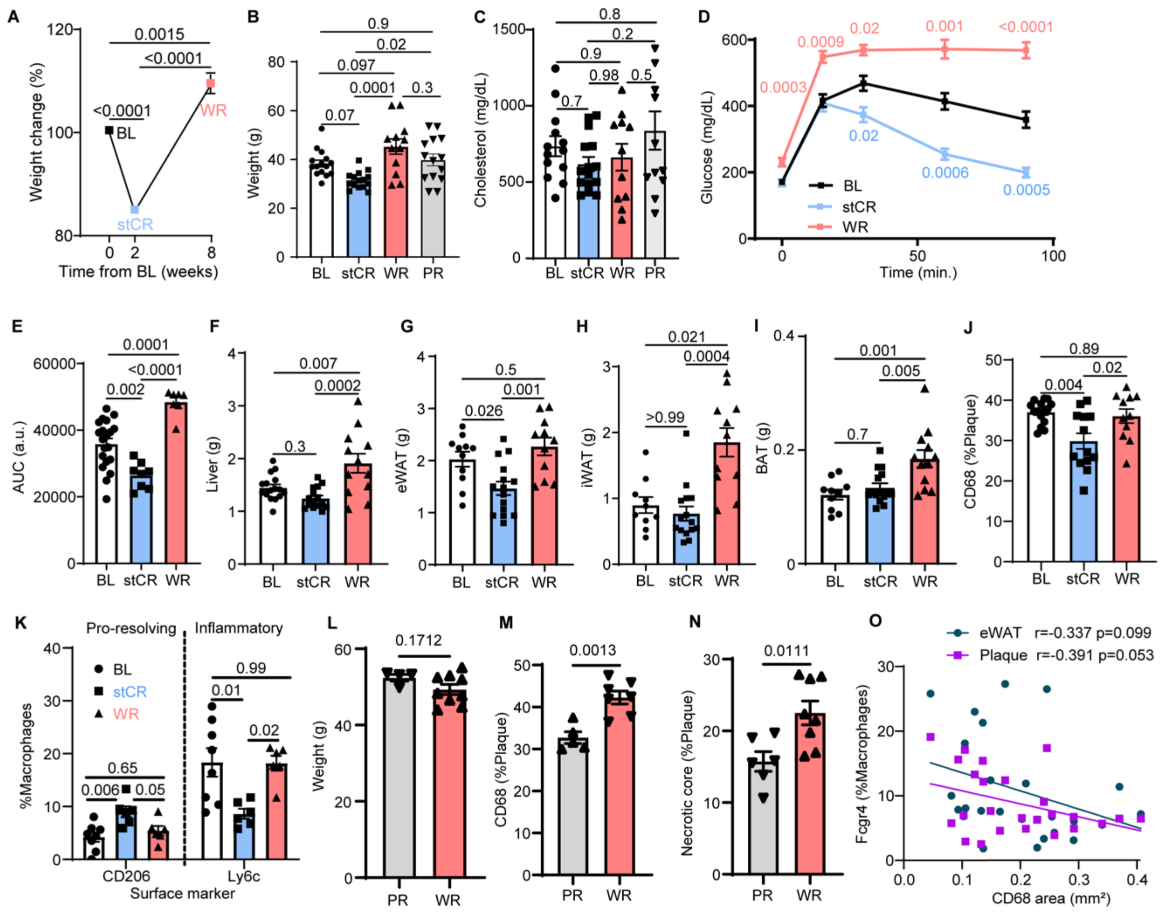

Supp. Fig. 5: Weight regain worsens metabolic parameters and plaque inflammation.

(A) Percent body weight change during weight cycling, n=29. (B) Body weight and (C) plasma cholesterol levels of all groups at harvest. (D, E) Glucose tolerance test performed in baseline, stCR and weight regain groups, n=7-20. (F-I) Tissue weights at the end of the experiment: (F) liver, (G) eWAT, (H) iWAT, (I) BAT. (J) Macrophage percentage of total plaque area measured in aortic root sections. (K) Flow cytometry analysis of aortic arch macrophages. (L) Body weights (M) CD68 and (N) necrotic core content in a separate cohort of mice harvested after 3 weeks of weight regain and

compared to mice that did not weight cycle (n=6-8). (O) Simple linear regression showing correlation between *Fcgr4*+ macrophages (Fcgr4 on Y-axis) from eWAT and plaques with total macrophage content in plaques. P-values were determined via (A-G, K) one-way ANOVA with Tukey's multiple comparisons test, (H) Kruskal-Wallis with Dunn's multiple comparisons test, (L-M) student t-test and (N) simple linear regression analysis. Data are shown as the mean ± SEM.

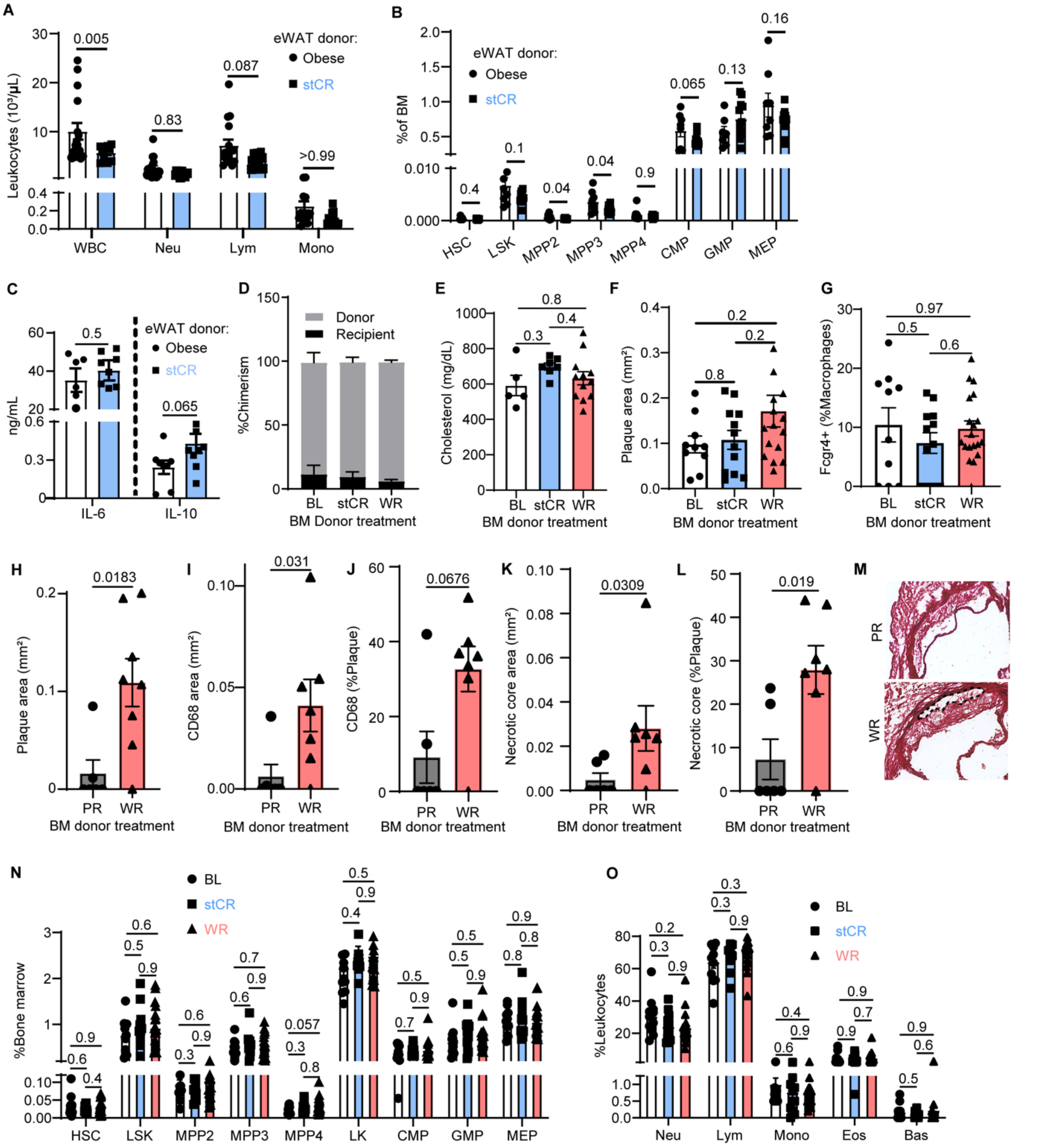

Supp. Fig. 6: Weight cycling induces long-term reprogramming of hematopoietic progenitors.

(A) Abundances of circulating white blood cells and (B) their bone marrow progenitors in mice transplanted with eWAT from obese and stCR donors (Fig. 4A). (C) Cytokines produced by bone marrow cells harvested from mice transplanted with obese or stCR

eWAT and treated ex vivo with LPS for 16h, n=6-8. (D) Engraftment frequencies of donor cells following BM transplantation, n=6-8. (E) Plasma cholesterol at the end of BM transplantation experiment, n=5-10. (F) Total plaque area of mice transplanted with BL, stCR and WR bone marrows and fed HFHC diet for 14 weeks. (G) Flow cytometry analysis of FCGR4+ macrophages in plaques, n=8-14. (H-M) Bone marrow transfer studies were repeated, with donors either continuously fed ad libitum (PR), or subjected to the weight cycling protocol (as in Fig. 6D; n=6-7). Recipient mice were harvested after 14 weeks of HFHC diet feeding and aortic root sections assessed for (H) plaque area, (I-J) macrophage content and (K-M) necrotic core content. (N) Frequencies of bone marrow hematopoietic stem and progenitor cells and (O) circulating white blood cells were analyzed at the end of the bone marrow transplantation experiment. P-values were determined via (A, B, O) two-way ANOVA with Sidak multiple comparison test, (C) two-tailed Student's t-test, (J-N) Mann-Witney test, and (E-G) one-way ANOVA with Tukey’s multiple comparisons test. Data are shown as the mean ± SEM.

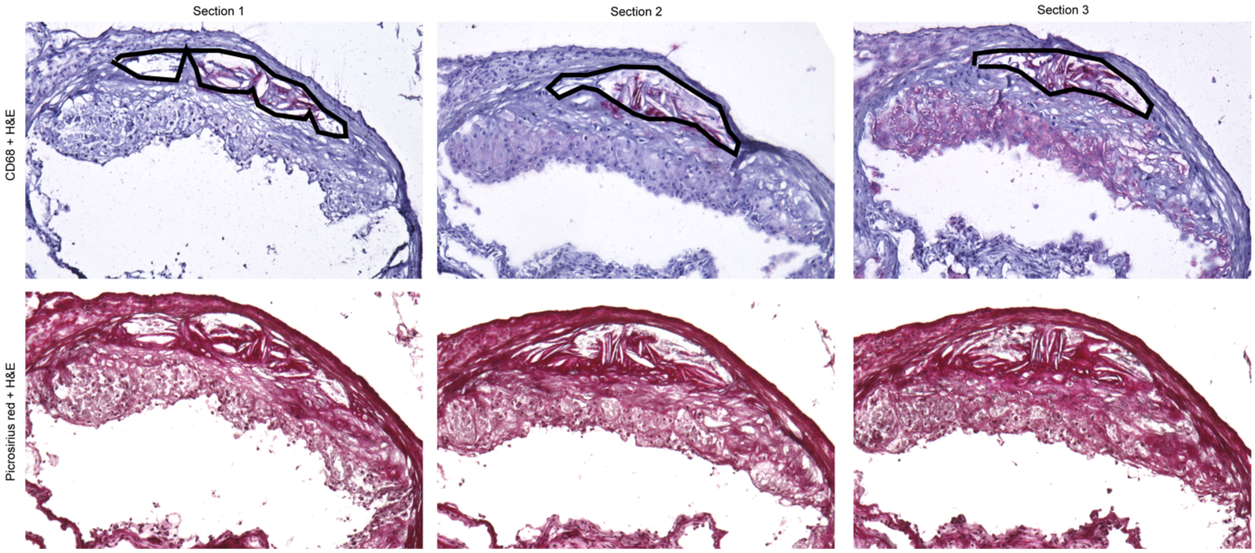

Supp. Fig. 7: Necrotic core quantification.

Adjacent sections (6μm apart) of aortic roots were stained for H&E together with either CD68 (top row) or picrosirius red (bottom row). Areas lacking both nuclei and extracellular matrix were defined as necrotic core (outlined in black). The picrosirius red images were used to confirm the necrotic core area selection in the CD68-stained slides, then used for quantification. Serial sections spanning >400μm were quantified per mouse. Data are shown as the mean ± SEM.
